# Construction of a CCL20-centered circadian-signature based prognostic model in cervical cancer

**DOI:** 10.1186/s12935-023-02926-6

**Published:** 2023-05-15

**Authors:** Yuchong Yu, Yao Liu, Yuhong Li, Xiaoming Yang, Mi Han, Qiong Fan

**Affiliations:** 1 Department of Gynecologic Oncology, The International Peace Maternity and Child Health Hospital, School of Medicine, Shanghai Jiao Tong University, Shanghai, China; 2 Shanghai Municipal Key Clinical Specialty of Gynecologic Oncology, Shanghai, China; 3 Shanghai Key Laboratory of Embryo Original Diseases Affifiliated to Shanghai Jiao Tong University School of Medicine, Shanghai, China

**Keywords:** Cervical cancer, Circadian clock, Prognostic model, Bioinformatics, Immunology

## Abstract

**Background:**

Rather low vaccination rates for Human papillomavirus (HPV) and pre-existing cervical cancer patients with limited therapeutic strategies ask for more precise prognostic model development. On the other side, the clinical significance of circadian clock signatures in cervical cancer lacks investigation.

**Methods:**

Subtypes classification based upon eight circadian clock core genes were implemented in TCGA-CESC through k-means clustering methods. Afterwards, KEGG, GO and GSEA analysis were conducted upon differentially expressed genes (DEGs) between high and low-risk groups, and tumor microenvironment (TME) investigation by CIBERSORT and ESTIMATE. Furthermore, a prognostic model was developed by cox and lasso regression methods, and verified in GSE44001 by time-dependent receiver-operating characteristic curve (ROC) analysis. Lastly, FISH and IHC were used for validation of CCL20 expression in patients’ specimens and U14 subcutaneous tumor models were built for TME composition.

**Results:**

We successfully classified cervical patients into high-risk and low-risk groups based upon circadian-oscillation-signatures. Afterwards, we built a prognostic risk model composed of GJB2, CCL20 and KRT24 with excellent predictive value on patients’ overall survival (OS). We then proposed metabolism unbalance, especially for glycolysis, and immune related pathways to be major enriched signatures between the high-risk and low-risk groups. Then, we proposed an ‘immune-desert’-like suppressive myeloid cells infiltration pattern in high-risk group TME and verified its resistance to immunotherapies. Finally, CCL20 was proved positively correlated with real-world patients’ stages and induced significant less CD8^+^ T cells and more M2 macrophages infiltration in mouse model.

**Conclusions:**

We unraveled a prognostic risk model based upon circadian oscillation and verified its solidity. Specifically, we unveiled distinct TME immune signatures in high-risk groups.

**Supplementary Information:**

The online version contains supplementary material available at 10.1186/s12935-023-02926-6.

## Background

With advancement in HPV preventative vaccines development, HPV positive cervical cancer (HPV^+^ CC), accounting for about 95% of CC, have been considered as preventable for HPV naïve young women [1]. However, HPV is not the only high-risk factor of CC, with approximately 5.5–11% CC HPV negative accompanied by poor prognosis [1]. Besides, HPV vaccines could not cover all HPV types, even for Gardasil-9 with expanded coverage to HPV types (6, 11, 16, 18, 31, 33, 45, 52, and 58) [2, 3], and large amounts of older women with pre-existing HPV infections remain unsuitable for such vaccines since they are not therapeutic [4]. On the other hand, late-stage CC diagnosed as distant or metastatic have limited treatment approaches and poor 5-year OS of only 20% [5]. Considering the rather low vaccination rates worldwide and large potential risk groups with HPV infection, gene signatures with prognosis and predictive value are in urgent need for CC patients.

Circadian clock refers to an oscillation pattern of divergent gene networks that cooperate to adapt to environmental cycles and internal timing system on a 24-h-basis. Its core regulators include an activator comprised of CLOCK and BMAL1 and a repressor comprised of Per1/2 and CRY1/2 [6]. Emerging evidence have indicated circadian rhythm as an indispensable factor for pathophysiological processes, such as in lifespan extension through caloric restriction and tumorigenesis. Recent literatures proved that chronic jetlag and chronic circadian disruption could accelerate breast tumor growth by creating an immune-suppressive TME [7]. Furthermore, CRY1/2-/- and Per1/2-/- knockout mice experienced enhanced hepatocarcinoma metastasis along with disrupted serum bile acids and glycogen metabolism [8]. On the other hand, CLOCK mutation mice exhibited resistance to carcinogen-induced skin carcinoma development [9], and knockdown BMAL1 by shRNAs also interrupted the proliferation of leukemia stem cells [10]. Pan-cancer analysis of TCGA provided transcriptional-level evidence of the heterogeneric effects of circadian clock related gene signatures in caner hallmarks as well. PER2 was found to be highly associated with activation of PI3K/AKT oncogenic pathway and cancer metabolism, while CLOCK was quite the opposite [11]. Although disruption of circadian rhythm has been proved to be strongly correlated with cancer hallmarks including uncontrolled proliferation, genome instability, deregulation of metabolism and immune-suppressive TME, the translational value remained obscure until recently. Jiao Wang et al. designed a time-restricted regimen, that is, giving combination therapy of metformin and trastuzumab at zeitgeber time (ZT) 6 rather than ZT18, which significantly suppressed breast tumor growth compared to trastuzumab alone in murine models [12]. To be noticed, such regimen failed to restrain tumor growth given at any other ZTs. They suggested that the underlying mechanism was that metformin-targeted HK2 fell into a circadian oscillation regulated by PPARγ and PER1 and disruption of HK2 interfered with such circadian rhythm thus leading to reversion of trastuzumab [12].

Recently, another circadian clock core gene, TIMLESS was found to be over-expressed in ovarian cancer (OV) and was negatively correlated with B cells and DC cells infiltration in TCGA data analysis [13]. Knockdown of TIMELESS significantly slowed tumor growth in vivo. While disruptions in circadian clock have been described in other gynecologic neoplasms, there have not been systematic bioinformatics analysis to date on circadian machinery of cervical cancer. Considering heterogeneity effects of circadian rhythm in cancer biology, knowledge of its functioning specifically in CC might benefit a lot.

Herein, to facilitate understanding into clinical relevance of circadian clock related signature in cervical cancer and illuminate its impact upon cancer metabolism and TME, we investigated TCGA-CESC dataset from the perspective of circadian oscillation thoroughly on a multi-omics level. Through K-means method, Cox regression and lasso analysis, we constructed a prognostic risk model based upon circadian-oscillation-signature and confirmed its validity by time-dependent ROC analysis. Furthermore, we discovered an ‘immune-desert’-like TME pattern in high-risk group by ESTIMATE and CIBERSORT analysis. Afterwards, to infer differences on genomic level, we conducted investigation upon SNP, CNV and TMB as well. With TIDE score and GDSC database, we then analyzed different drug sensitivity, including to immune checkpoints blockades (ICB), in high and low-risk groups. Then in combination with clinicopathological features, we built a risk prognostic model based upon independent prognostic factors and verified its solidity in GSE44001.Finally, through FISH and IHC analysis of our own real-world patients’ clinical surgery specimens, we validated CCL20 as an independent indicator for worse stages, and overexpression of CCL20 in U14 cell lines could lead to significant more M2 macrophages, Tregs and less M1 macrophages, in accordance with CIBERSORT analysis.

## Methods

### Data downloaded and arrangement

The cervical cancer dataset TCGA-CESC was obtained from the TCGA-GDC official website (https://portal.gdc.cancer.gov/). Gene expression sequencing data (counts and FPKM values) (n = 307) were then transformed into TPM values separately. Clinical data of patients, including age, gender, TNM stage, survival time, and survival status, were downloaded. After excluding patients who lacked clinical information, 304 samples with clinical information were retained. The clinical data of the patients can be found in Additional file 1: Table S1. Genomics sequencing data of CESC patients (n = 304) were downloaded from GDC, among which masked somatic mutation data were selected and visualized using the maftools R package. Validation data were obtained from GEO (https://www.ncbi.nlm.nih.gov/geo/) under the accession number GSE44001. Normal cervical tissue samples were also collected from GTEx, GSE173097, GSE55940, GSE9750, GSE127265 and GSE20167. For immunotherapy analysis, datasets in GSE78220, GSE91061, GSE93157, GSE94873, GSE111636, GSE123728, GSE165252 and GSE176307 were analysed. In order to deal with the imbalance between normal(13) and tumor sample (304) numbers in TCGA, we adopted ROSE R package [14], which has been widely used, with ovun.sample function with parameters as follows: p = 0.5, method = "under". Chip platform was based on GPL14951 Illumina HumanHT-12 WG-DASL V4.0 R2 expression beadchip. Finally, 300 tumor samples were retained for inclusion in this study, and the chip data were standardized using the R limma [15] package. The circadian rhythm-related gene set was obtained from the previously published literature [1]. which contains a total of 24 genes, including ARNTL, ARNTL2, CLOCK, CRY1, CRY2, PER1, PER2, PER3, TIMELESS, BHLHE41, BHLHE40, CSNK1D, CSNK1E, DBP, FBXL3, HLF, NFIL3, NPAS2, NR1D1, NR1D2, RORA, RORB, RORC, and TEF. The different expression genes (DEGs) between high-risk and low-risk groups were compared by Wilcoxon rank-sum test and visualized through the pheatmap R package.

### Subtype classification and analysis based on circadian rhythm gene signatures

Based on circadian rhythm genes and TCGA-CESC expression data, an unsupervised cluster analysis was performed using the "K-means method" in the "ConsensusClusterPlus" R package [16] with parameter distance of “Pearson” to identify circadian rhythm subtypes in cervical cancer patients. The consensus clustering algorithm was used to determine the number of clusters, and the analysis included 1000 iterations to ensure the stability of the classification. Principal component analysis (PCA) was performed on subgroups to judge the differences between samples. Survival analysis was performed after grouping to determine impact of grouping on prognosis.

### Differential and prognostic circadian rhythm-related genes screening and risk model construction

The Wilcoxon rank-sum test and sleuth [17] R package was used to analyze the differences between different groups, genes with significant differences were defined as the absolute value of Log2 (Fold change) > 1.0 and adj.P-value < 0.05. in order to obtain differential circadian rhythm-related genes.To dig for the prognostic value of circadian rhythm-related genes in CESC patients, univariate Cox analysis was first used to screen for prognostic genes, with a threshold of P-value < 0.1. We used lasso and multivariate Cox proportional hazards regression model to identify independent prognostic factors further and establish a prognostic model. We then used tenfold cross-validation to test the model. The formula for calculating the risk score of the risk model is as follows:

$${\text{risk}}Score = \mathop \sum \limits_{i} Coefficient \left( {gene_{i} } \right)*mRNA Expression \left( {gene_{i} } \right)$$

Patients were divided into high-risk and low-risk groups based on risk scores. ROC curves for 1, 3, and 5 years were plotted by time-dependent ROC curves to determine their accuracy. In addition, the model was tested using the external test set GSE44001 according to the regression coefficients of the genes in the model, and a time-dependent ROC curve was drawn for validation.

### DEGs and functional enrichment analysis of circadian rhythm-related signatures

To identify DEGs correlated with the circadian rhythm-related-risk model built above, sleuth [17] and Wilcoxon rank-sum test was used to decipher DEGs between the separated groups. The significantly DEGs were defined as the absolute value of logFC > 1 and adj.P-value < 0.05.Gene ontology (GO) analysis is a standard strategy for pathways enrichment studies, which include biological process (BP), molecular function (MF), and cellular component (CC) [18]. The Kyoto Encyclopedia of Genes and Genomes (KEGG) is a basic method adopted to induce enrichment pathways, such as those related to genomes, biological pathways, and drug metabolism [19]. ClusterProfiler package[20] was used to perform GO and KEGG analysis, and a cutoff value of FDR < 0.05 was considered statistically significant. To investigate the specific pathway pattern between different groups, we performed GSEA (Gene Set Enrichment Analysis) analysis [21]. "C2.cp.v7.2.symbols.gmt" and "C5.all.v7.2.symbols" were downloaded from MSigDB [22] database (https://www.gsea-msigdb.org/gsea/msigdb/index.jsp) for GSEA analysis. FDR < 0.25 was considered to be included.

### Protein interaction and regulatory network analysis

The STRING protein–protein interaction database was used to analyze the interaction between differential genes. The core hub genes were further explored through the CytoHubba plugin in Cytoscape [23]. The hub gene-ceRNA regulation analysis was performed using the mirTarbsse database (mirtarbase.cuhk.edu.cn). The results were filtered based on the experimentally verified results in the luciferase reporter assay. Results were finally visualized by Cytoscape software.

### Identification and correlation analysis of tumor infiltrating immune cells

The immune landscape of the tumor microenvironment (TME) was assessed using the R ESTIMATE package [24]. ESTIMATE analysis quantifies immune activity (level of immune cells infiltration) in tumor. Differences in infiltration characteristics were further compared between high and low-risk groups. Subsequently, to determine the specific level of immune cells subtypes infiltration, we deconvolved the transcriptome expression matrix using the CIBERSORT algorithm [25] (https://cibersortx.stanford.edu) to estimate the composition and abundance of different immune cells. ggplot2 R package was used to show the distribution of 22 immune cell infiltrations in TCGA-CESC dataset.

### Single nucleotide polymorphism (SNP) and copy number alteration (CNV) analysis

To analyze SNPs we used maftools package to analyze frequently mutated genes in patients in high and low-risk groups. Subsequently, patients' masked copy number segment data were downloaded through GDC and subjected to GISTIC 2.0 analysis [26] by GenePattern [27] with default parameters as follows: t_amp = 0.1, t_del = 0.1, join_segment_size = 4,qv_thresh = 0.1,remove_X = 1,res = 0.05,conf_evel = 0.75,do_gene_gistic = 0,do_arbitration = 1,arm_peeloff = 0,sample_center = median.

### Tumor mutation burden (TMB), microsatellite instability (MSI) and predictive analysis of tumor immunotherapy

We calculated TMB by maftools R package. The MSI-Sensor data of CESC patients were obtained from the cBioportal database (https://www.cbioportal.org). In addition, we predicted the potential response of ICB through the Tumor Immune Dysfunction and Exclusion (TIDE) score (http://tide.dfci.harvard.edu) [28] between the high and low risk groups.

### Drug sensitivity analysis

The Genomics of Drug Sensitivity in Cancer (GDSC) database (www.cancerrxgene.org/) can be used to find tumor drug response data and sensitive markers [29]. We used the pRRophetic algorithm [30] to construct a ridge regression model based on gene expression profiles and predicted the sensitivities of high-risk and low-risk groups to common anticancer drugs through IC50 values.

### Construction of clinical prediction model based on circadian rhythm risk score

To demonstrate the independent predictive value, we used univariate Cox and multivariate Cox to analyze the predictive power of risk score combined with clinicopathological features of patients as regards to overall survival (OS). We constructed a clinical prediction model, resampling it with the bootstrap method for validation. According to the clinical significance and statistical value, the clinical prediction nomogram (Nomogram) was constructed. To quantify discriminative performance, time-dependent ROC curves for 1, 3, and 5 years were plotted. A calibration curve was generated to assess the performance of the nomogram by comparing the predicted values of the nomogram with the observed actual survival data.

### *Fluorescence *in situ* hybridization (FISH) analysis of formalin-fixed, paraffin-embedded (FFPE) tissue samples*

22 patients’ cervical cancer surgery specimens were included in this study in the International Peace Maternity and Child Health Hospital, School of Medicine affiliated to Shanghai Jiao Tong University. All patients had stage I/II CV without disease progression except for one patient. In situ hybridization was carried out using the RNAscope fluorescent multiplex assay (Advanced Cell Diagnostics) After dehydration the sections were incubated with pretreat 4 for 20 min at room temperature and hybridized with probes for CCL20 mRNAs for 2 h. CCL20 mRNA copy numbers were determined by quantification of fluorescent spots using ImageJ software.

### Immunohistochemistry (IHC) analysis of FFPE tissue samples

FFPE sections from the biopsies were subjected to IHC by using multiplex IHC kit (Panovue, Beijing, China, Cat No 0004100100). Briefly, primary antibodies were sequentially incubated followed by horseradish peroxidase (HRP)-conjugated secondary antibody and a tyramidefluorophore (Panovue), Nuclei were stained with 4`-6`-diamidino-2-phenylindole (DAPI) (SIGMA-ALDRICH, MI, USA) before the observation.

### Overexpression of ccl20 in U14 cell lines and flow cytometry analysis of subcutaneous mouse tumor model

U14 cells were purchased from the Shanghai Institute for Biological Sciences Chinese Academy of Sciences (Shanghai, China) and routinely maintained in DMEM with 10% fetal bovine serum (FBS) and 1% Penicillin–Streptomycin (Gibco, grand Island, US, Cat No 15140-122) at 37℃ with 5% CO2. C57BL/6 mice (6–8 weeks old) were purchased from the Shanghai Laboratory Animal Center (Shanghai, China) and maintained under specific-pathogen-free (SPF) conditions in the animal facility of SJTU School of Medicine (SJTUSM). For the sake of animal ethics guidance, the number of mice in each mouse cage did not exceed 5.In order to obtain reliable survival data of mice, up to 33 mice were breeded in multiple cages at different floors of our animal centre. U14 were infected with lentivirus containing plasmid (pcSLenti-EF1-mCherry-P2A-Puro-CMV-ccl20-3xFLAG-WPRE) at the multiplicity of infection (MOI) of 1:70 and 4 μg/mL polybrene (Hanbio, Cat No HB-PB-500) for 24 h. After 48 h, cells were cultured with 2 μg/mL puromycin (Sangon Biotech, Shanghai, China, Cat No A610593) for 14 days. Afterwards, monoclonal cell population was constructed by limited dilution within 21 days and chosen by qRT-PCR and western blot verification. Mice were subcutaneously injected with 0.5 × 10^6^ U14 cells [31]. At day21, mice were euthanasia by Carbon dioxide (CO^2^) inhalation, and tumors were harvested and digested for flow cytometry analysis with antibodies as follows: CD45-Apc-cy7 (BD, cat: 557659), CD4-Bv786 (BD, cat: 563331), CD3-Bv496 (BD, cat: 564661), CD8-Bv650 (BD, cat: 563234), MHC-II- percp-cy5.5 (BD, cat: 562363), CD11b-Af700 (BD, cat: 557960), F4/80-Bv605 (BD, cat: 743281), live/dead- Bv510 (BD, cat: 564406), Foxp3-pe-cy7 (ebioscience, cat: 25-5773-82), CD25-pe-cf594 (BD, cat: 562694), CD206-Bv421 (Biolegend, cat: 141717), CD86 PE (BD, cat: 564198). 12 mice every independent experiment for three times were sacrificed. Single cells were acquired with a Fortessa flow cytometer (BD Biosciences). Data were analyzed by using FlowJo software 9.0 (FlowJo LLC, Treestar Inc., OR, USA). The protocols of animal experiments were approved by the Animal Ethics Committee of SJTUSM, and performed under the Guide for the Care and Use of Laboratory Animals.

### Statistical analysis

**Fig. 1:**
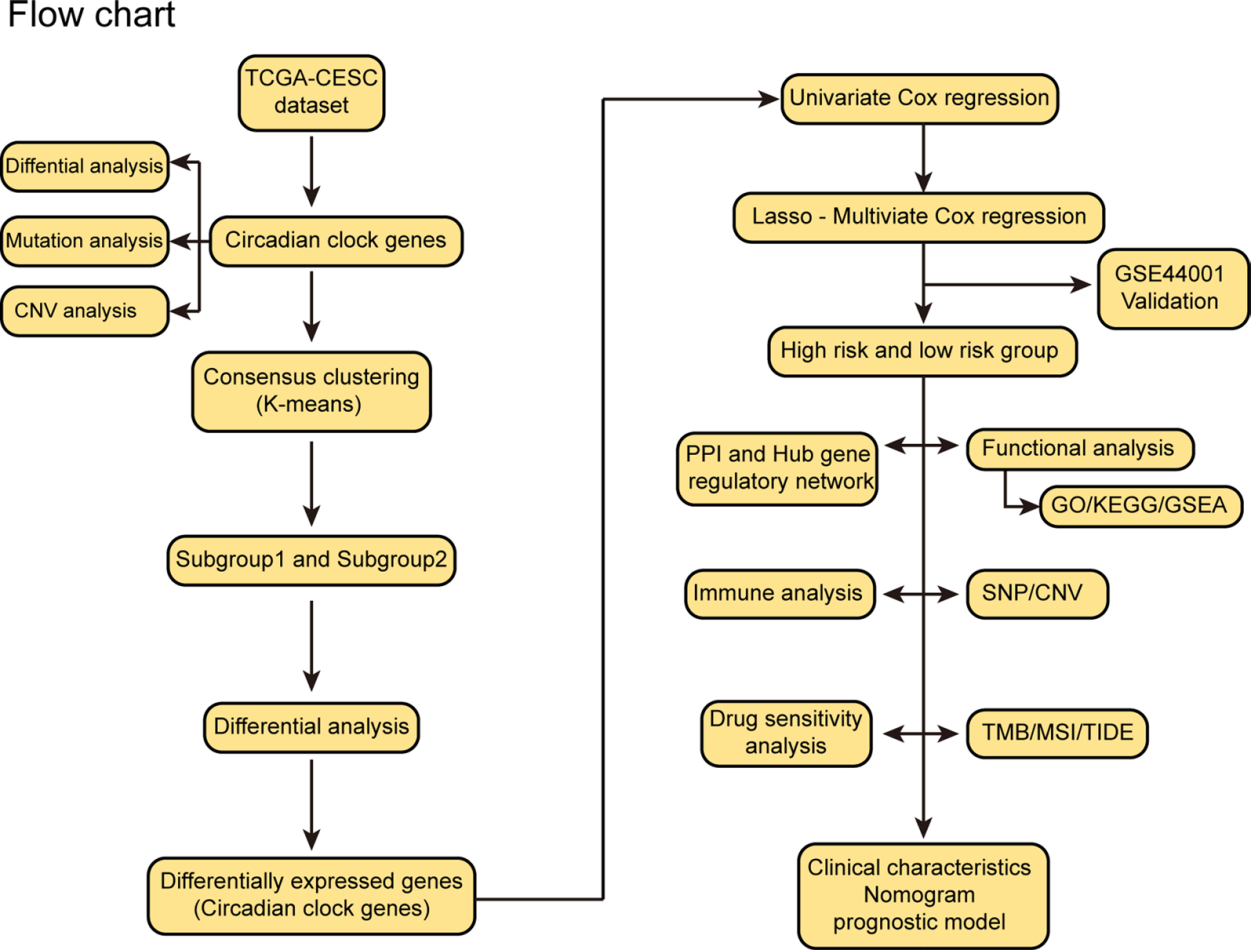
Flow chart

All data processing and analysis were implemented through R software (version 4.1.3). To compare two groups of continuous variables, the statistical significance of normally distributed variables was estimated by the independent Student t-test. The differences among non-normally distributed variables were analyzed by the Mann–Whitney U test (i.e., the Wilcoxon rank-sum test). The Chi-square test or Fisher's exact test was used to compare and analyze statistical significance between two groups of categorical variables. The survival R package was used for survival analysis. The log-rank test was used to evaluate the significance of the difference in survival time between the two groups. A time-dependent receiver operating characteristic (ROC) curve was drawn using the pROC package for R, and the area under the curve (AUC) was calculated to assess the accuracy of the risk model in predicting prognosis [18]. All statistical P values were two-sided, with P < 0.05 considered statistically significant (Fig. 1).

## Results

### Integrated transcriptome and genome analysis of circadian rhythm genes in multiple databases revealed differences between tumor and normal cervical tissues

**Fig. 2:**
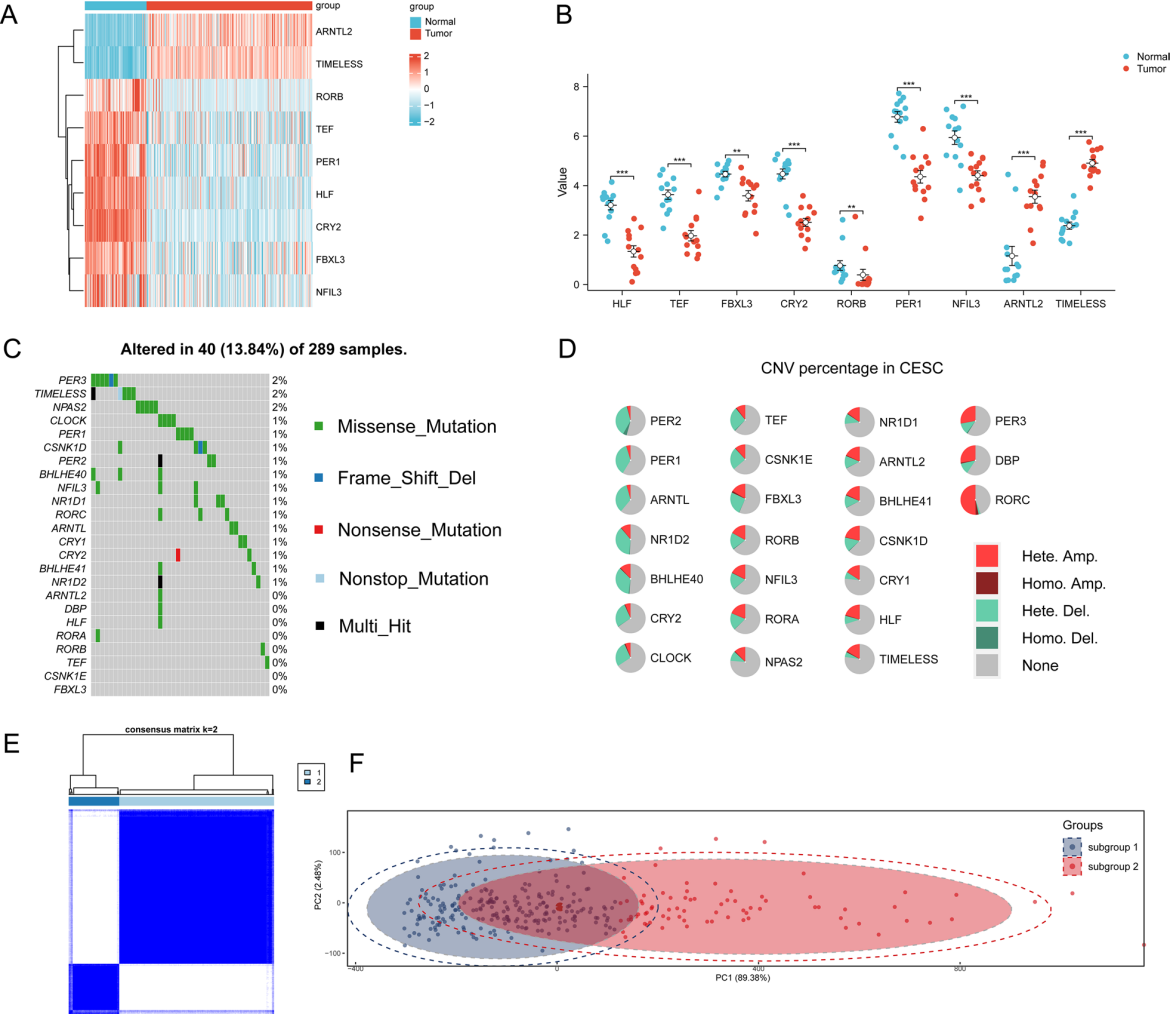
Differences in circadian gene expression and subtype identification in CESC patients. **A** heat map of differential expression of circadian rhythm genes in normal group (number = 114) and tumor group (number = 304); **B** representative dotplot of differential expression of circadian rhythm genes in normal group (number = 13) and tumor group (number = 15) with resampling method in ROSE R package (described in methods in detail); **C** is the somatic mutational change of circadian rhythm genes in CESC patient group; **D** pie chart shows the CNVs of 24 circadian rhythm-related genes in CESC; **E** is the heat map of sample clustering when K = 2 using consistent clustering; **F** is the PCA map of subgroup 1 and subgroup 2

Since Youqiong Ye et al. investigated circadian clock core genes expression and mutation landscape across pan-cancer TCGA database except for CESC, we firstly extracted 24 circadian rhythm genes from the RNA-seq data of TCGA-CESC and compared the expression differences between normal and tumor groups. In comparing the normal and tumor groups, eight circadian rhythm genes were differentially expressed, including HLF, TEF, FBXL3, CRY2, RORB, PER1, NFIL3, ARNTL23, and TIMELESS (Additional file 1: Fig. S1A, B). Since there were only 13 in TCGA-GTEx normal cervival tissues, we collected normal cervical tissues from different GEO databases, and compared these differentially expressed circadian genes between GEO-collected and GTEx normal tissues and there was no difference (Additional file 1: Fig. S1C upper). Then, we compated their expression between all normal tissues and TCGA tumor samples (Fig. 2A and Additional file 1: Fig. S1C lower) and use resampling methods only in TCGA-GTEx samples (Fig. 2B) separately, and the conclusion remained the same. Finally, we used GSE9750 as validation, and the expression differences were in accordance with analyses mentioned above (Additional file 1: Fig. S1D). For example, CLOCK showed no difference between normal and tumor samples while ARNTL2 was significantly up-regulated in tumor samples (Additional file 1: Fig. S1D). Then, we extracted the mutation information of 24 circadian rhythm gene and found that the circadian rhythm genes did not have obvious mutations in CESC patients based on the somatic mutation data of TCGA-CESC patients. The mutation frequency of most of these genes is less than 1%. The mutation frequency of PER3, TIMELESS, and CLOCK genes is only 2%, and they have not been reported to be associated with CESC (Fig. 2C). In addition, we analyzed the CNV alteration patterns of 24 genes (Fig. 2D), the details of which are shown in Additional file 1: Table S2. Although amplification and deletion appeared frequently in TCGA-CESC, CNV pattern varies across different circadian clock core genes. For example, heterozygous amplification is dominant in RORC, while heterozygous deletion in PER1/2 (Fig. 2D).

Due to the low mutation rate and heterogeneous CNV pattern of circadian clock genes in TCGA-CESC, we then focused on the differentially expressed circadian genes between tumor and normal mentioned above and used consensus clustering as the clustering strategy. Since k = 2 is sufficient for well-separated subgroups, we separated patients into subgroup 1 and subgroup 2 (Fig. 2E), the apparent difference between which were implicated by PCA analysis as well (Fig. 2F).

### Construction of circadian rhythm signature-derived risk model uncovered GJB2, CCL20 and KRT24 as independent prognosis indicators

**Fig. 3:**
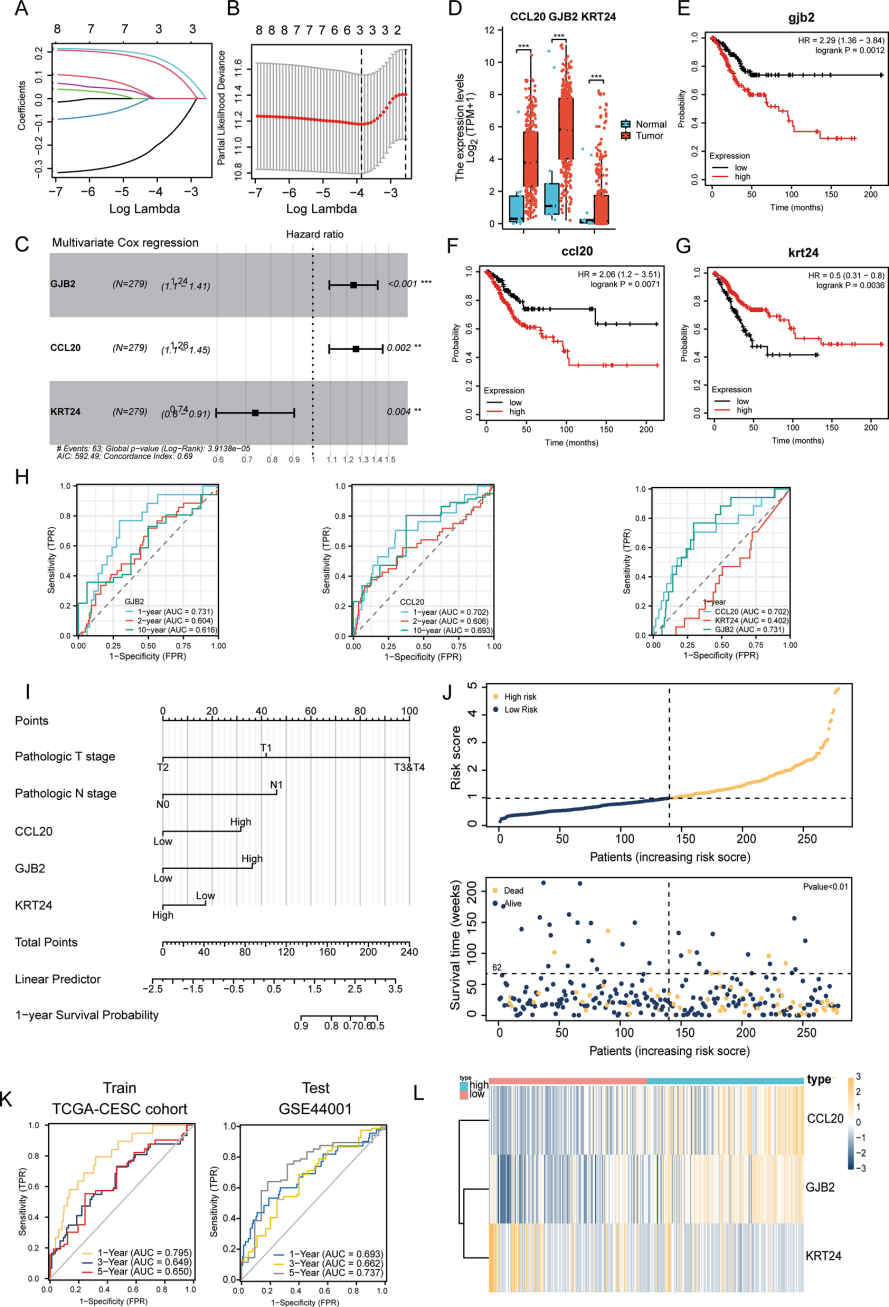
Construction of a circadian rhythm-related risk scoring model. **A**–**B** are LASSO regression analysis, and the number of variables corresponding to the optimal λ value is 3. **C** is the result of multivariate Cox stepwise regression analysis, and finally found that all three genes are independent prognostic factors; **D** is the expression of KRT24, CCL20 and GJB2 in normal group (number = 114) and tumor group (number = 304); **E**–**G** are the survival curves of KRT24, CCL20 and GJB2 with Kaplan–Meier analysis in TCGA-CESC data (number = 304); **H** are time-dependent ROC curve analysis of KRT24 (left) and CCL20 (middle) in TCGA-CESC data, with the one on the right as the 1-year ROC curve of KRT24, CCL20 and GJB2; **I** is the prognostic nomogram of pathologic T, N stages and KRT24, CCL20, GJB2 in TCGA-CESC; **J** is the risk score distribution of CESC patients, the survival status of patients; **K** are time-dependent ROC curve analysis of the training set TCGA-CESC (left), and the right is the test set GSE44001; **L** is heat map of KRT24, CCL20 and GJB2 expression in high-risk (number = 124) and low-risk groups (number = 180)

In order to dampen our understanding of these two subgroups, we used the DESeq2 package for differential analysis and finally got 86 differential genes (logFC absolute value > 1, adj. P < 0.05) (Additional file 1: Fig. S1E, F). Then, we integrated these significantly differentially expressed circadian rhythm-related genes to construct a circadian rhythm-related risk scoring model to quantitatively evaluate the prognostic information of each CESC patient by risk score. First, a univariate Cox regression analysis was performed, and eight qualified genes were selected for further research (P < 0.05). Furthermore, through lasso analysis we found that when λ = 3, we could build the most solid model (Fig. 3A, B). Afterwards, by multivariate Cox regression analysis, we found that GJB2 (P < 0.001), CCL20 (P = 0.002), and KRT24 (P = 0.004) were all independent prognostic factors separately (Fig. 3C).

GJB2 (Gap Junction Protein Beta 2) (also named CX26) encodes a member of the gap junction protein family. To be noticed, CX26 enhance the self-renewal potency of triple-negative breast cancer stem cells through formation of protein complex with the pluripotency transcription factor NANOG and focal adhesion kinase (FAK) [31]. CCL20 (C–C Motif Chemokine Ligand 20) is exclusively overexpressed by myeloid-derived suppressive cells (MDSC). Since its major receptor CCR6 is highly expressed on T cells, researchers have illuminated its participation in suppressive TME [32]. KRT24 (Keratin 24) belongs to the type I (acidic) keratin family, which involves in the intermediate filament (IF) formation. There still lacks research about KRT24 in cancer biology till now.

Afterwards, we checked their expression between normal and tumor samples, and found out that they were all significantly up-regulation in tumor (Fig. 3D). Besides, in line with the Cox regression analysis, GJB2 and CCL20 is indicative of worse overall survival rate (OS), and KRT24 is beneficial (Fig. 3E–G). To construct a multivariate cox regression-based risk model, we examined their saparate time-dependent ROC analysis firstly. GJB2 and CCL20 were all excellent indicator for prognosis prediction while KRT24 alone was rather general for 1-year OS (Fig. 3H, I). On another way, we conducted time-dependent AUC analysis for KRT24, and found out that its protective value for survival was more obvious for 5-year OS (Additional file 1: Fig. S2A) and progression free survival (PFS) overall (Additional file 1: Fig. S2B, C). Considering its significant prognostic value and prediction value in PFS, we also included KRT24 in the riskscore model. Based on the penalty coefficients of feature genes derived in multivariate cox regression analysis, a risk score was calculated by cumulative sum of multiplication of gene expression with corresponding coefficients, and then risk score of each sample was calculated. Time-dependent ROC analysis showed that the risk score had good predictive power for OS in CESC patients, with the areas under the curve (AUC) for 1-, 3-, and 5-year OS of 0.795, 0.649, and 0.650, respectively (Fig. 3K left). For verification, we selected dataset GSE44001, normalized and further tested performance of the risk model. Time-dependent ROC analysis in verification dataset showed that AUC for 1-year, 3-year, and 5-year PFS was 0.693, 0.662, and 0.737, respectively, indicating excellent reliability and stability of our risk model (Fig. 3K right). In addition, significant worse prognosis of CESC patients was observed along with gradually rising risk score (Fig. 3J, Additional file 1: Fig. S3B). Finally, GJB2 and CCL20 showed significant more expression in high-risk group which is in accordance with their prognosis value (Fig. 3L).

### Immune and metabolism-related pathways were enriched in DEGs analysis between high and low-risk groups derived from circadian rhythm signature-related risk model

**Fig. 4:**
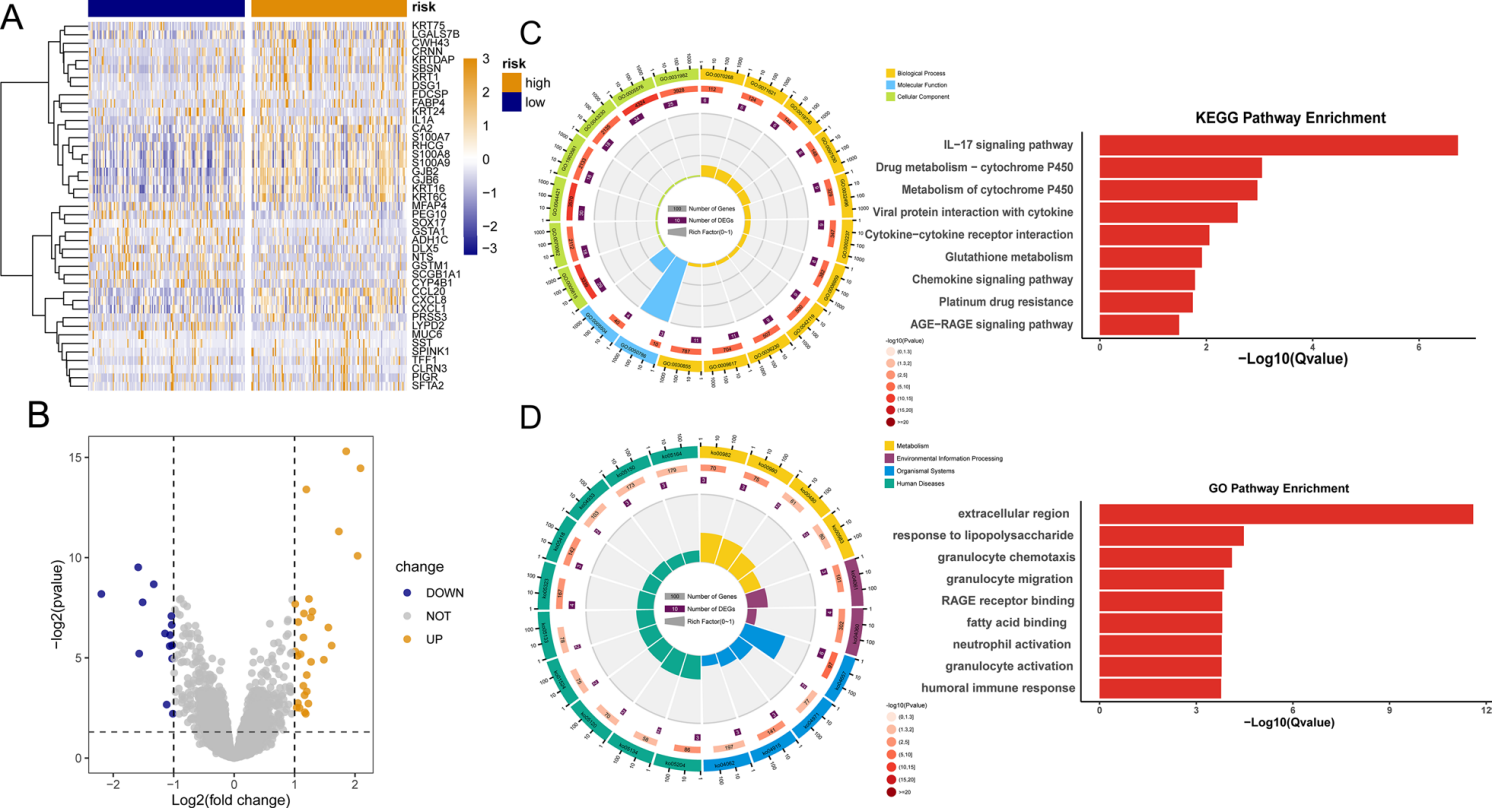
Analysis of differentially expressed genes and functional enrichment based on circadian rhythm-related risk models. **A**–**B** are volcano plots and heat maps showing the expression of DEGs between high and low risk CESC patients in the TCGA dataset; **C** is GO analysis suggesting differential genes and response to lipopolysaccharide, response to molecule of bacterial origin, epithelial cell differentiation. It is related to biological processes such as antimicrobial humoral response, and is related to molecular functions such as RAGE receptor binding and fatty acid binding, and is related to cell composition such as extracellular region and extracellular region part; **D** is the result of KEGG analysis showing that these differentially expressed genes are involved in IL-17 signaling pathway, Drug metabolism–cytochrome P450 and other pathways. Pathways in **C** and **D** all have qvalues less than 0.05

In order to further explore the impact of circadian rhythm-related gene signatures on CESC, patients were divided into high-risk and low-risk groups based on the median expression risk scores. Subsequently, we performed differential expression analysis and found 43 significantly differentially expressed genes (DEGs), of which 29 genes were significantly up-regulated and 14 genes were significantly down-regulated (Figs. 4A, B).

Afterwards, we performed functional enrichment analysis of 43 DEGs. GO analysis showed enrichment of biological processes such as response to lipopolysaccharide, epithelial cell differentiation, and antimicrobial humoral response, and also molecular functions such as RAGE receptor binding, fatty acid-binding, and extracellular region (Fig. 4C). Interestingly, the independent prognosis factors we discovered above, KRT24 and GBJ2, all participate in formation of cell junction and extracellular matrix. Besides, KEGG analysis indicated that DEGs mainly enriched in IL-17 signaling pathway, drug metabolism-cytochrome P450, and Rheumatoid arthritis (Fig. 4D), together with GO analysis indicating potential immune participation.

Then we thoroughly went through GO and KEGG common results and unveiled a significant enrichment of pathways related to immunology, such as IL-17 signaling pathway, chemokine signaling pathway, and also pathways correlated to metabolism, such as glutathione metabolism. Detailed GO and KEGG results can be found in Additional file 1: Tables S3 and S4.

**Fig. 5:**
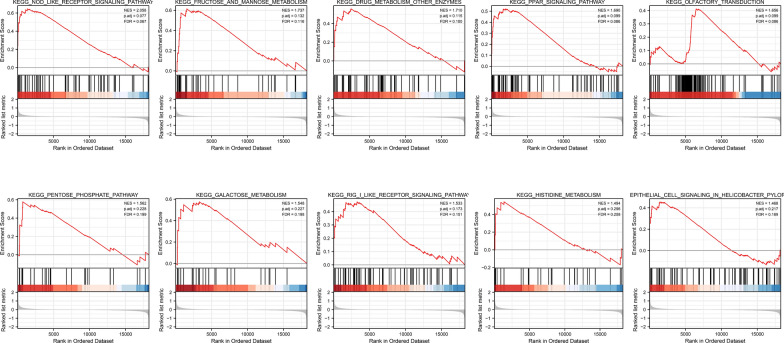
GSEA analysis. The main pathways enriched in patients are shown from the rank of low-risk to high-risk groups (left to right) according to the enrichment score NES value

To facilitate and verify GO and KEGG analysis, we conducted GSEA analysis based upon DEGs. It appeared that NOD-like receptor signaling pathway, RIG -like signaling pathway, fructose and mannose metabolism, pentose phosphate pathway, galactose metabolism, PPAR signaling pathway, and other pathways are enriched in low-risk group (Fig. 5). The nucleotide oligomerization domain (NOD)-like receptors 1 and 2 (NOD1/2) are intracellular pattern-recognition proteins that activate innate immune signaling pathways [33], and RIG-like receptors cooperate with Toll-like (TLR) receptors to impart innate and adaptive immune response as well [34, 35]. Apparently, GSEA analysis unveiled immune and metabolism centered pathways enrichment either, although specific pathways differ from GO and KEGG analysis. Detailed GSEA enrichment results are shown in Additional file 1: Table S5.

### PPI network and non-coding RNA analysis found CCL20-centered hub_genes

**Fig. 6:**
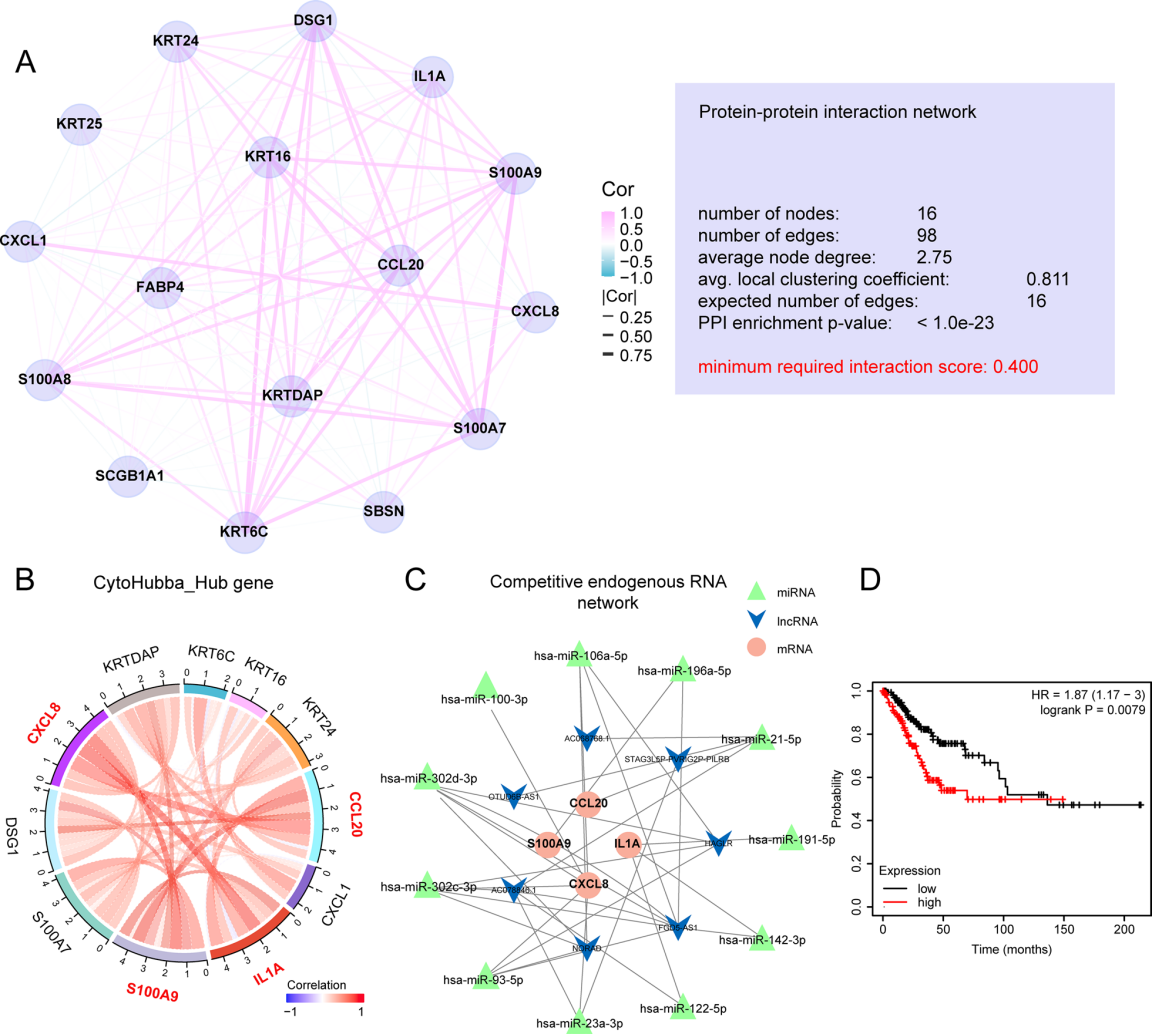
PPI and regulatory network analysis. **A** is the PPI control network that is re-analyzed from the most specific co-interacted network in Fig. S4A, which shows the node information, connecting line information and different sub-network information of the network in detail. **B** is the hub gene regulation network based on CytoHubba calculation. **C** is the miRNA-lncRNA-ceRNA regulatory network predicted by the mirTarbase database; **D** is the survival plot of patients with different expression of hsa-mir-302d and has-mir-196a, with the high group representing patients with high mean expression of hsa-mir-302d and has-mir-196a in TCGA-CESC (number = 304)

With enrichment analysis pointing to immune and metabolism related pathways, to further narrow down our investigation of DEGs to a set of core hub genes, we used STRING database to analyze the protein interaction pattern (Additional file 1: Fig.S4). With confidential threshold set to 0.4, the interaction pattern of DEGs could be further divided and clustered into four sub-interaction networks. To further concentrate on the core valuables in the PPI network, we chose the network with the most candidate proteins (Fig. 6A). The number of new PPI nodes (proteins) is 16, and there are 98 connecting lines (edges). The average connection degree of each node is 0.811, and the enrichment statistical P-value of the entire PPI is less than 1.0e-23. Since certain genes centered CCL20 having the most connecting edges, we set out to hypothesis that they might serve important functions.

Subsequently, we used the CytoHubba plugin in Cytoscape to further identify which interacting proteins could be core hub genes. After calculation, ten hub proteins with the highest scores, including CCL20, CXCL1, S100A9, and CXCL8, were found (Fig. 6B). Next, we analyzed the miRNAs molecules and lncRNAs potentially regulating these hub genes through the mirTarbase database and used Cytoscape to construct a ceRNA regulatory network (Fig. 6C). Astonishingly, CCL20, CXCL8, S100A9, and IL1A are all tightly correlated with myeloid immune cells chemotaxis and functioning [32, 36–39], and long non-coding RNAs, HAGLR and FGD5-AS1 are related to suppressive immune TME either [40, 41]. To facilitate better understanding into this protein-non-coding RNAs interation network, we checked their prognostic values in TCGA-CESC miRNA database, and found out that hsa-mir-302d and has-mir-196a in this network were indicative of worse prognosis (Fig. 6D).

### Analysis of immune infiltration revealed a “cold” TME in high-risk gorup

**Fig. 7:**
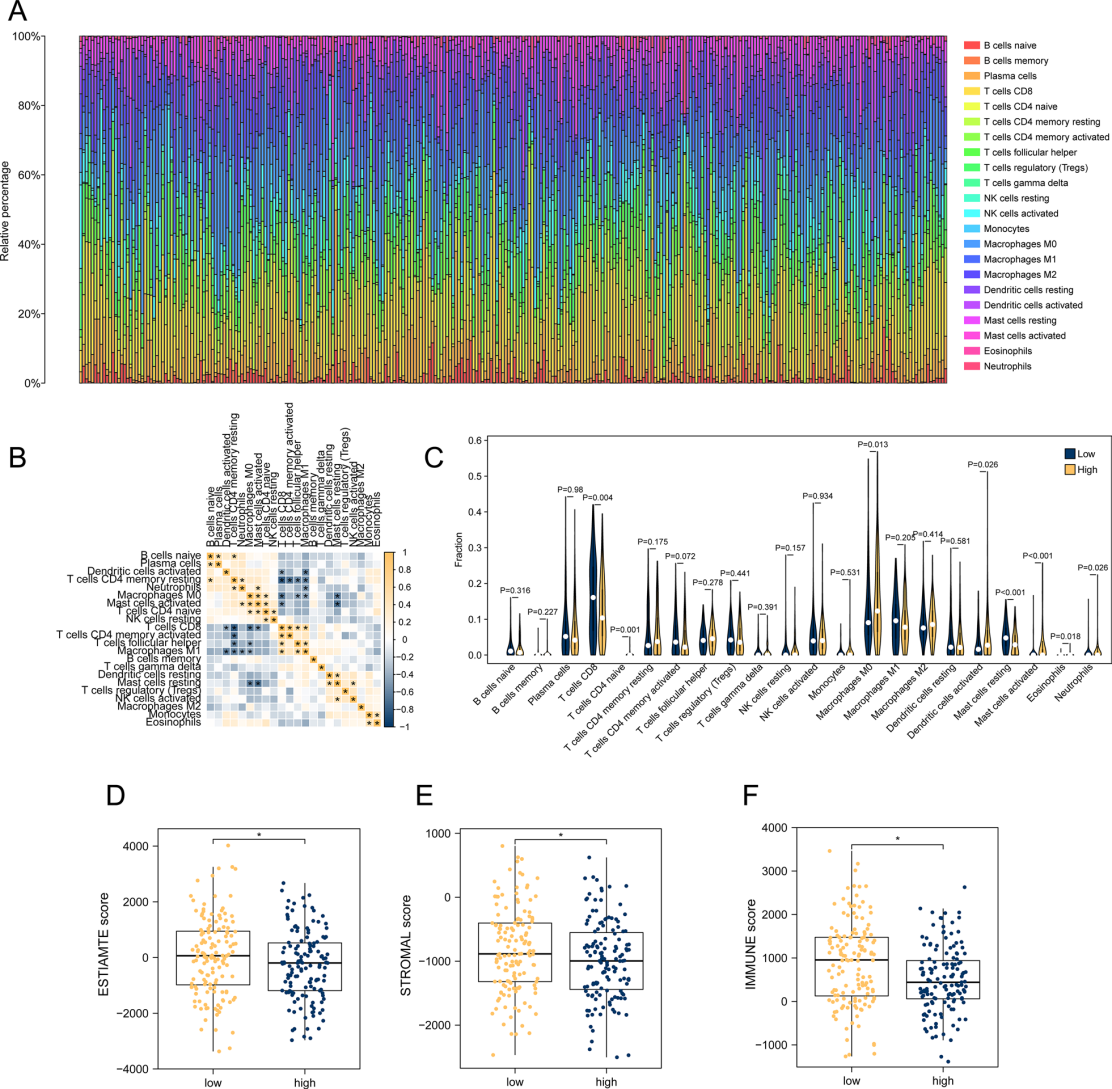
Association of circadian rhythm-related risk scores with different immune cell infiltrations. **A**–**B** are the panorama analysis and immune cell correlation analysis of immune cell infiltration in the whole CESC patients. **C** is the difference analysis of 22 different immune cell infiltration levels between the two groups (number in high-risk group = 124, in low-risk group = 180); **D**–**F** is the difference of Estimate score, stromal score and immune score in high and low risk groups. *P < 0.05, **P < 0.01, ***P < 0.001, ****P < 0.0001

As a result, we assessed the effect of circadian rhythm-related risk scores on the overall immune profile in TCGA-CESC patients. It could be noticed that distribution of immune cells in CESC patients is heterogeneous, reflecting the complexity of the TME (Fig. 7A). The correlation analysis showed significant negative correlation between CD8 T cells, M0 macrophages (M0), activated mast cells and activated DCs (Fig. 7B). At the same time, CD8 T cells showed significant positive correlation with M1 macrophages (M1), activated memory CD4 T cells (activated CD4 Tm) and CD4 T follicular helper cells (CD4 Tfh). Afterwards, when comparing the infiltration of immune cells in the high-risk group versus the low-risk group, we also discovered significant more infiltration of CD8 T cells (P = 0.004) and an increasing trend of infiltration of activated CD4 Tm (P = 0.072) in low-risk group. Besides, high-risk group could be characterized as TME with significant more M0 (P = 0.013), more neutrophils (P = 0.02), more activated DCs (P = 0.026) and more activated mast cells (P < 0.01) (Fig. 7C), in accordance with correlation analysis. In conclusion, low-risk TME is tend to be one with ‘hot’ tumor features such as CD8 T cells and activated CD4 Tm, while high-risk TME is more likely to be one with suppressive myeloid cells infiltration pattern such as M0 and neutrophils.

Furthermore, according to the ESTIMATE results, compared with the low-risk group, patients in the high-risk group had significantly lower levels of cell infiltration abundance, fewer stromal cells and immune cells as well (Fig. 7D–F). Together with the correlation analysis and immune cells subtypes analysis, we suggested that high-risk group has higher tumor purity and an ‘immune-desert’-like TME with significant more suppressive myeloid cells infiltration than low-risk group.

### Effect of circadian rhythm-related risk score on genomic changes in CESC patients showed genome instability in high-risk group

**Fig. 8:**
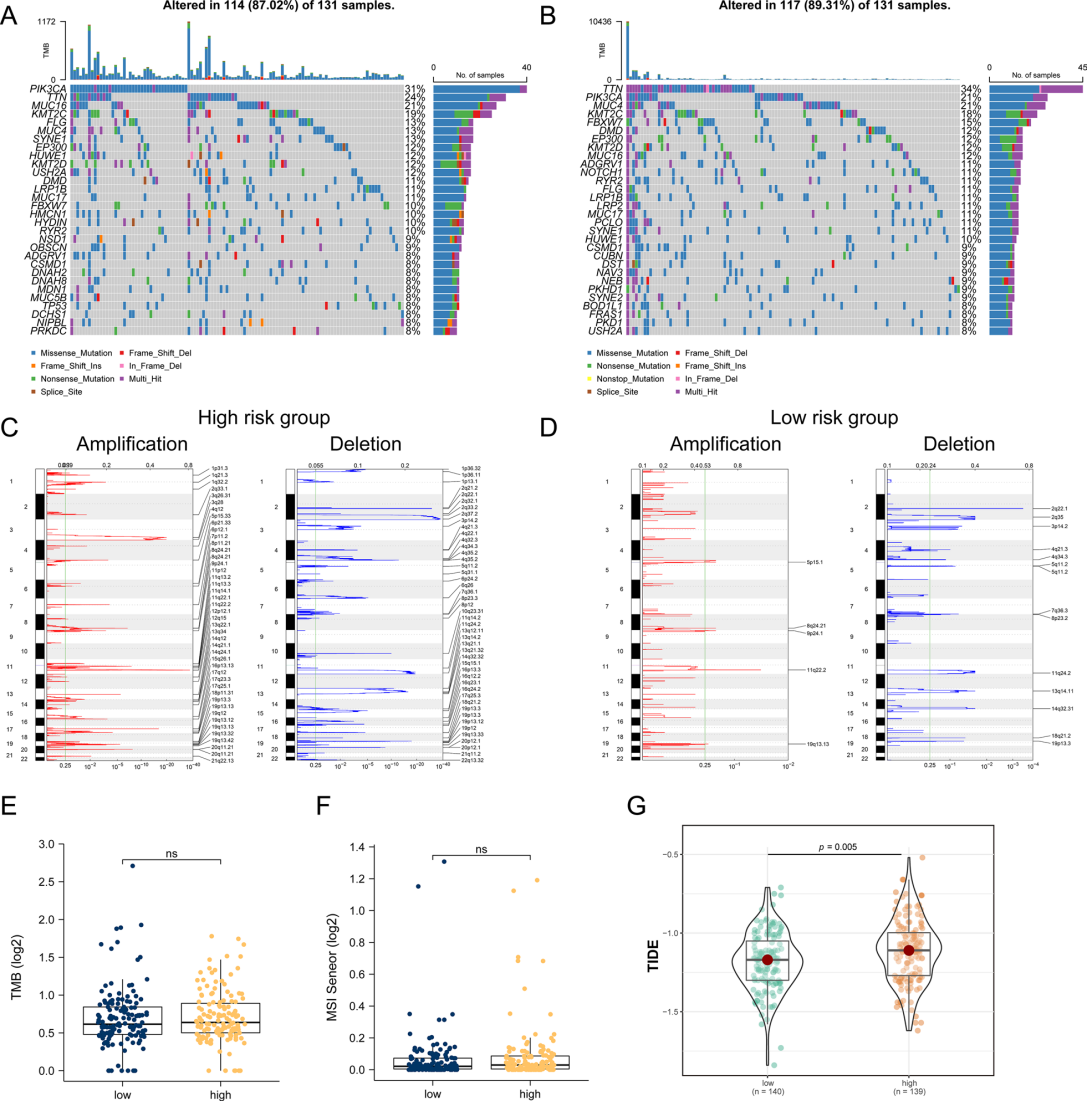
The effect of circadian rhythm-related risk grouping on genetic variation and immunotherapy in CESC patients. **A**–**B** are mutational profiles of common tumorigenesis driver genes in high- and low-risk groups of patients. Mutation information for each gene in each sample is shown in a waterfall plot, with various colors indicating different mutation types; subsections above the legend show mutational load; **C**–**D** are changes in copy number levels of different genes in patients in high and low risk groups, in which red is the gene with significantly increased copy number, and blue is the gene with significantly deleted copy number; **E**–**F** are the differences in TMB and MSI levels of patients in the high and low risk groups, respectively; **G** is the TIDE score calculated based on the TIDE database Differences in high and low risk groups

However, whether genome changes also participated in changes among high and low-risk groups remained to be solved. As has been indicated in 2.1, circadian clock genes themselves did not show significant genomic level changes between subgroups in CESC. We further assessed the effect of circadian rhythm-related risk scores on genomic level in CESC patients, including single nucleotide polymorphisms (SNP) and copy number variations (CNV). Analysis of SNPs in driver oncogene mutations revealed that both groups of patients have similar alterations (Fig. 8A), while CNV of both amplification and deletion were more frequent in high-risk group (Fig. 8C, D). When it comes to TMB and MSI, we did not find substantial differences between high and low-risk groups, suggesting that changes in the genomic level were not evident (Fig. 8E, F).

In addition, given the critical role of cancer immunotherapy, we evaluated sensitivity to immunotherapy by TIDE scores. Surprisingly, TIDE score was significantly lower in high-risk group than in low-risk group, indicating worse responsiveness of immunotherapy of high-risk group, which was in accordance with its ‘immune-desert’-like suppressive myeloid infiltrated TME mentioned above.

### High-risk group tended to be heterogeneous and have worse immunotherapy effects

**Fig. 9:**
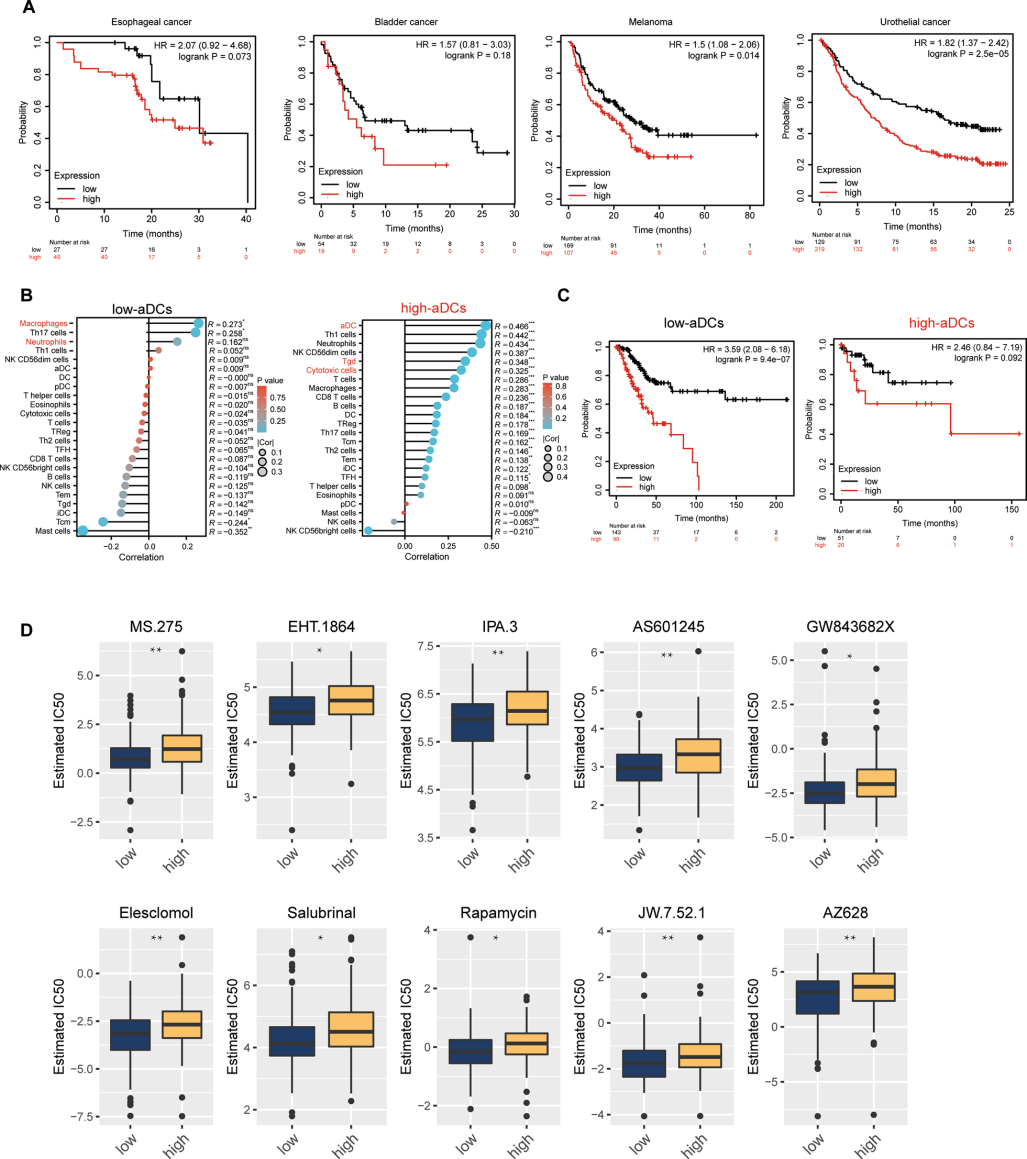
Immunotherapy prognosis and drug sensitivity analysis of circadian rhythm-related high- and low-risk groups. **A** are the survival plots of patients separated by the risk model in different immunotherapy cohorts; **B** is ssGSEA analysis of immune infiltration patterns of different patients separated by CIBERSORT scores of aDC expression (low and high by mean expression); **C** is survival plot of patients separated by CIBERSORT scores of aDC expression in TCGA-CESC (number = 304); **D** is according to the wilcox test comparing the drug IC50 of high and low risk groups, the top 10 drugs with p value are MS.275, EHT.1864, IPA.3, AS601245, GW843682X, Elesclomol, Salubrinal, Rapamycin, JW7.52.1, AZ628. These drugs all showed higher sensitivity in the low-risk group than in the high-risk group. Statistical analyses in (D) were calculated by non-parametric student-t test analysis between low and high risk groups’ estimated IC50 values. *: P < 0.05; **: P < 0.01; ***: P < 0.001. *: P < 0.05; **: P < 0.01; ***: P < 0.001; ns: no significance

Since TIDE score indicated worse immunotherapy outcomes in high-risk group, we set out to examine the prognosis value of our risk-model in different immunotherapy cohorts. Surprisingly, our circadian genes-derived model showed excellent prediction value in different immune cohorts (Fig. 9A), especially in melanoma (P = 0.014) and urathelial cohorts (P = 2.5e-05). Unfortunaly, we did not find any cervical cancer immunotherapy cohorts. However, considering activated DCs (aDCs) were actually enriched in high-risk group while they indicated a somewhat “hot” TME on the opposite and aDCs varied greatly inside the high-risk group, we further separated patients in TCGA according to their activated DCs enrichment score calculated by CIBERSORT, and analysed their immune enrichment pattern by ssGSEA and prognosis (Fig. 9B, C). Astonishingly, patients with fewer aDCs have significant more macrophages and neutrophils, while those with higher aDCs have more gamma-delta T cells and cytotoxic T cells. In the low-aDCs group, our risk model was still solid, while its prediction power extremely vanished in the high-aDCs group (Fig. 9C), indicating therapy stratagies targeting DCs might be a way to overcome the worse prognosis of immunotherapy in the high-risk group.

Since we have uncovered certain immunotherapy resistance in the high-risk group, we then set out to assess differences in susceptibility to common antitumor drugs between high- and low-risk groups using the GDSC database (Fig. 9D). First, we use the GDSC data as the training set and the pRRophetic package to build a ridge regression model. Then, we input the TCGA-CESC dataset for testing. The test results found that among the 138 input drugs, 41 drugs were detected to be statistically different between the two groups (Additional file 1: Table. S6). Astonishingly, EHT.1864, IPA.3 and AS601245 are all more sensitive to low-risk groups, as these regimens are related to ATP-related pathways’ inhibition or competition, suggesting possible metabolic unbalance in high-risk groups as aforementioned above (Fig. 9D).

### Construction of a clinical prognostic model based on circadian rhythm-related risk scores

**Fig. 10:**
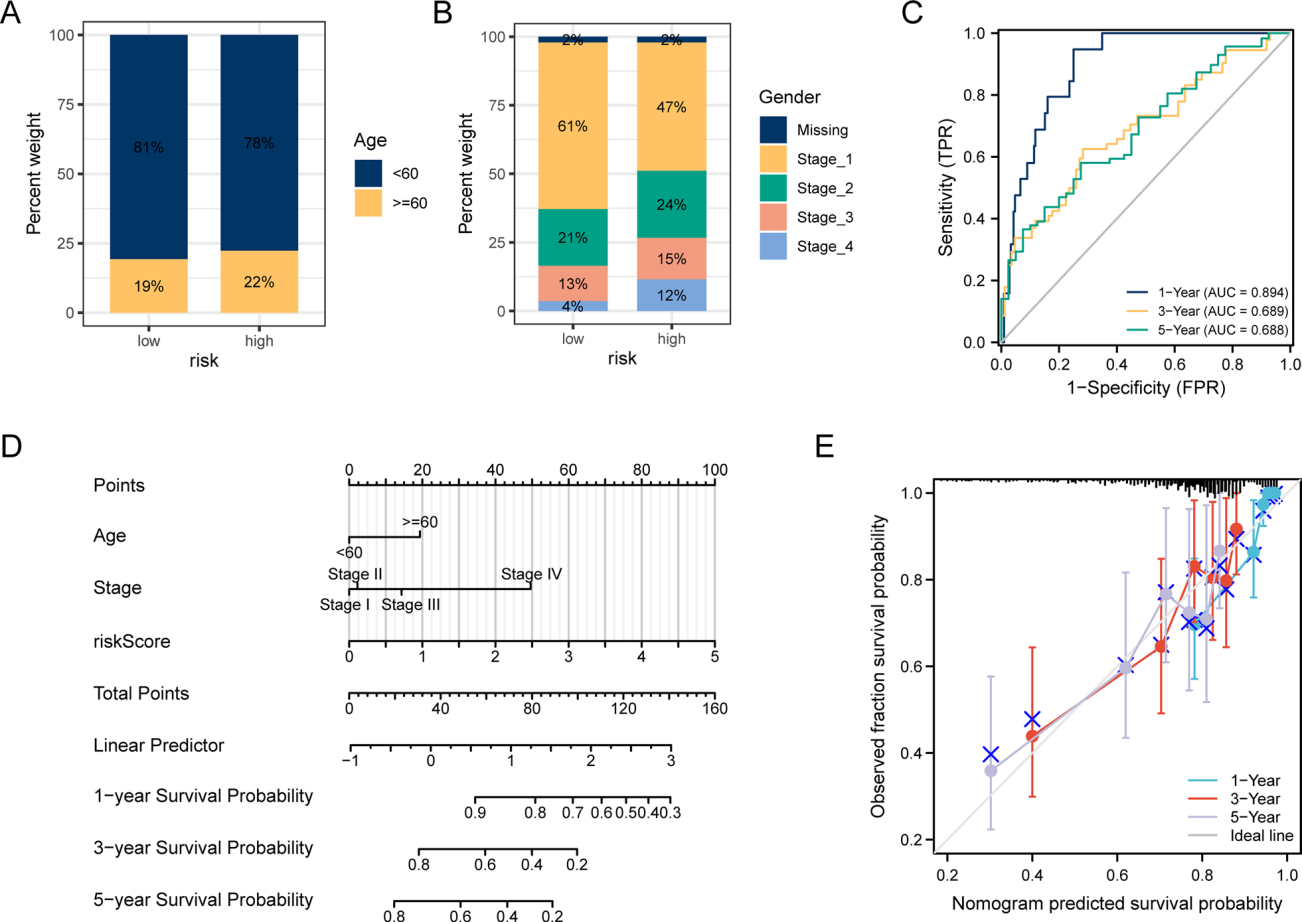
Analysis of the predictive power of circadian rhythm-related risk scores for prognosis in patients with CESC. **A**–**B** superimposed histograms show the proportion of age and stage of patients in high and low risk groups in the two groups. Age was similarly represented in both groups, with significantly more early-stage patients in the low-risk group than in the high-risk group. **C** is the time-dependent ROC curve of the riskscore-based clinical prediction model. **D** is the nomogram of the model. **E** is the calibration curve of the nomogram, using the bootstrap method and resampling 1000 times; the abscissa is the survival predicted by the nomogram, and the ordinate is the actual observed survival, repeated 1000 times each time, the curve shows the model pair Patient outcomes at 1, 3 and 5 years had good predictive value

Finally, to further explore the clinical value of circadian rhythm-related risk scores, we integrated patients’ clinical characteristics into our prognostic risk model. Although no difference was found in age (Fig. 10A), in terms of stage, the proportion of advanced patients (stage II, III and IV) in the high-risk group was significantly higher than that in the low-risk group (Fig. 10B). We then constructed a prognostic model based on the circadian rhythm-related risk score and clinicopathological characteristics (age and TNM stage) of CESC patients.We tested the model by resampling 1000 time (bootstrap method). It was found that 1-year, 3-year, and 5-year AUCs were 0.894, 0.689, and 0.688, respectively (Fig. 10C). We then visualized our model through nomogram, drew a calibration curve to evaluate the model's accuracy, and found that its 1-, 3-, and 5-year OS estimates showed excellent consistency with the actual observations (Fig. 10D). Finally, except for KRT24, all features chosen showed significant values in the multivariate analysis both for OS and disease free survial (DSS) (Additional file 1: Fig. S3C, D).

### CCL20 as independent indicator for worse stages in real-world patients and less functional CD8^+^ T cells with more M2 infiltration in mouse tumor models

**Fig. 11:**
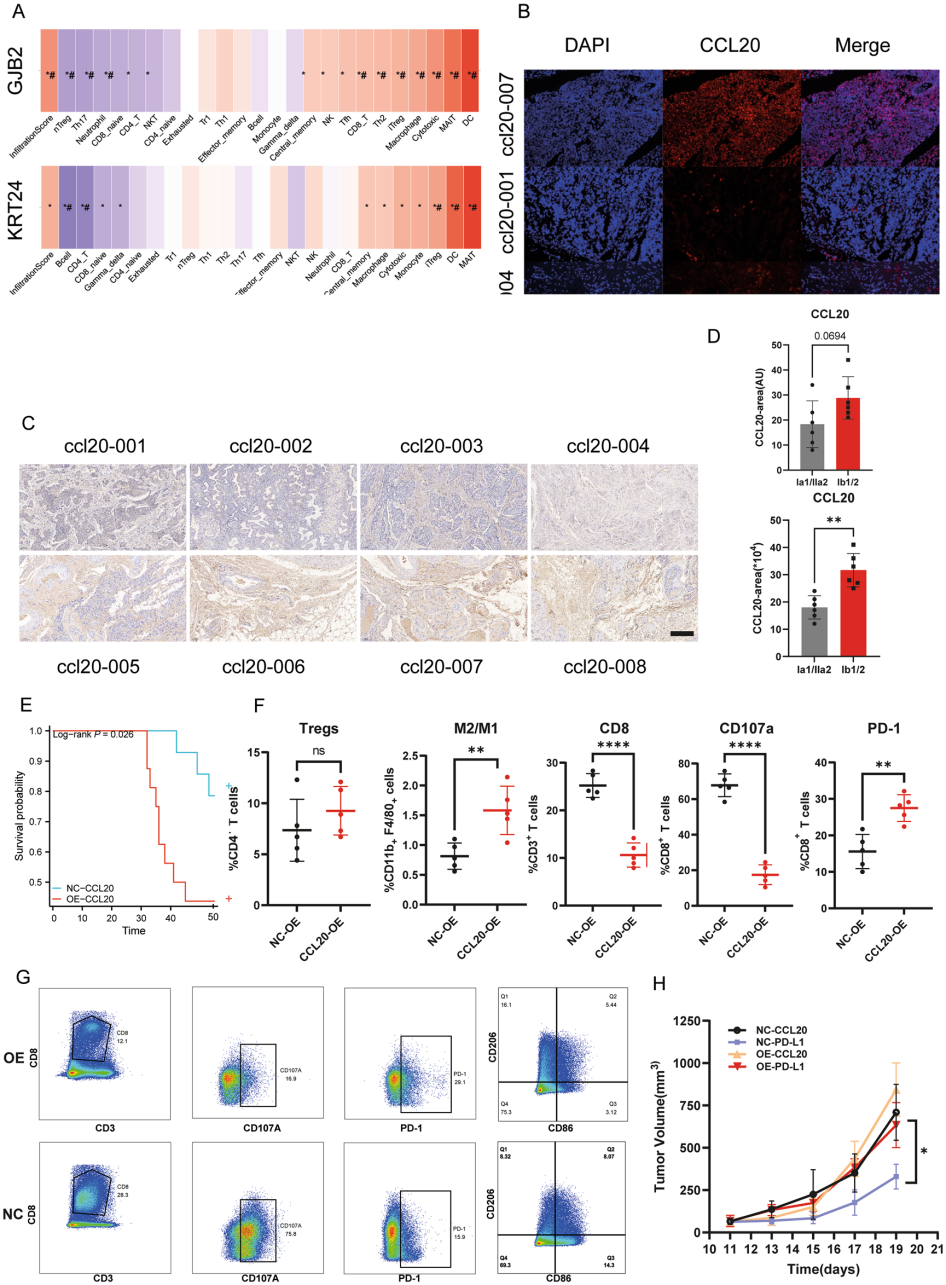
CCL20 as an independent indicator for worse prognosis and capable of enhancing M2 macrophages infiltration. **A** GSVA score of different immune subtype cells infiltration seperately for GJB2, KRT24 and CCL20. **B** FISH analysis for CCL20, with different rows standing for different patients, as ccl20-007, ccl20-001 and ccl20-004 as three representative images. **C** IHC analysis for CCL20. **D** ImageJ quantitative analysis for FISH (up) and IHC (down). **E** is the survival plot of mice during observation of 55 days, with NC-CCL20 referring to mice inoculated with empty plasmid loaded lentivirus infected U14 cells as control and OE-CCL20 referring to mice inoculated with CCL20 overexpression U14 cells (mice in NC-CCL20 are 14, and OE-CCL20 are 17, which were pooled together in separate cages, with each having less than 5 mice); **F** FC analysis for Tregs, M2/M1, CD8, CD107a and PD-1 in TME of U14 C57BL/6 J mouse model; **G** are representative FC gating graphs for CD8, CD107a expression of CD8, PD-1 expression of CD8, and macrophages with CD86^+^ CD206^−^ as M1 and CD86^−^ CD206^+^ as M2; **H** is the tumor growth curve of mice with different treatments, with NC-PD-L1 referring to NC-CCL20 mice with PD-L1 blocking antibody(αPD-L1) treatments, OE-PD-L1 referring to OE-CCL20 mice with αPD-L1 treatments, NC-CCL20 and OE-CCL20 referring to mice with PBS treatments as control groups (mice in each group:NC-PD-L1: 6; OE-PD-L1:6; NC-CCL20:5; OE-CCL20: 5). The data were the representatives of at least three independent experiments as means ± S.E.M. Statistical analyses were calculated by non-parametric student-t test or two-sided ANOVA analysis. *: P < 0.05; **: P < 0.01; ***: P < 0.001. *: P < 0.05; **: P < 0.01; ***: P < 0.001; ns: no significance. Scale bar in **B**: 100 μm; **C**: 200 μm

Although the prognostic value separately for GJB2, KRT24 and CCL20 have been verified in our bioinformatics analysis of TCGA and GEO data, the exact biological and clinical value of them deserved further research. Through GSVA immune scores independently on GJB2, KRT24 and CCL20 (Fig. 11A), we found that role of GJB2 and KRT24 separately in CESC were rather obscure since they had ImmuneScore indicative of “hot” TME with cytotoxic immune cells and DC infiltration. On the other way, CCL20 tended to favor a “cold” TME with significantly less CD8 T cells, more macrophages and more Tregs, has been deciphered through CIBERSORT analysis mentioned above (Fig. 7). Then we set out to investigate the exact expression levels of CCL20 within real-world clinical patients’ data. We collected 22 early stages (Ia1/IIa2, Ib1/2) CESC patients’ surgical specimens and performed FISH for RNA quantification (Fig. 11B). To be noticed, ccll20-007 is the only patient that has recurrence of disease within 5 years, and CCL20 RNA was significantly up-regulated between ccl20-007 and ccl20-001 and ccl20-004, the other two both showed no recurrence. Besides, through IHC analysis for CCL20 protein expression (Fig. 11C), patients that has later stages (Ib1/2, lower panel) had significant more CCL20 expression compared to those at earlier stages (Ia1/IIa2). Quantification analysis of FISH and IHC (Fig. 11D). supported an enhanced CCL20 expression in later stages patients as well. Finally, we constructed lentivirus inducted stable overexpression of CCL20 in U14 (OE-CCL20), and verified the successful overexpression by qRT-PCR, western blot, flow cytometry and IHC (Additional file 1: Figure S5).Then subcutaneous U14 tumor models were established in C57BL/6 mouse. Although we did not obsereved faster tumor growth in CCL20-OE mice (Fig. 11H), we did find out a significant worse survival in CCL20-OE mice (Fig. 10E). By flow cytometry analysis of TME, elevated ratio of M2/M1 was manifested in CCL20 overexpression (OE) group (Fig. 11E, F), along with less CD8^+^ T cells, less CD107a expression and more PD-1 expression of CD8^+^ T cells, with Tregs of no significance, in accordance with CIBERSORT analysis (Fig. 10F, G).Finally, considering the significant worse prognosis in high-risk group in immunotherapy cohort, we conducted anti-PD-L1 therapy in CCL20-OE mice, and found the same immunotherapy resistance as well (Fig. 10H), further supporting the same “immune-dessert” TME in mice characterized by less CD8^+^ T cells and more M2 macrophages.

## Discussion

Cervical cancer is the fourth most common female cancer worldwide [5]. Besides, overall low prophylactic vaccination rates among adolescents worldwide and preexisting infections in older women demonstrate the urgent need for developing state-of-the-art early diagnostic strategies and therapeutic regimens [42], for example, immunotherapies. Circadian disruption has been associated with tumorigenesis through effects on tumor proliferation, DNA repair and stemness, and combination therapies targeting circadian disorders with other regimens are emerging for GC [12], NSCLC [43], etc. However, little is known about its effects in tumor metabolism and TME, especially in cervical cancer, as well as its prognostic and therapeutic value [44]. In this research, we systematically investigated circadian rhythm related genes genomic and transcriptional level patterns in TCGA-CESC dataset. We successfully divided CESC patients into low-risk and high-risk groups based upon their circadian clock gene signature expression modes. We further confirmed immune and metabolism related pathways enrichment of differentially expressed genes (DEGs) between high and low-risk groups, which includes response to lipopolysaccharide, fructose and mannose metabolism, and IL-17 signaling pathway. Furthermore, we also uncovered significantly different immune infiltration patterns between them. Through lasso regression analysis of DEGs, we proposed a prognosis model composed of GJB2, CCL20 and KRT24, all with independent prognostic value, and validated its predictive value on patients’ overall survival (OS) in dataset GSE44001. Finally, we illuminated that low-risk group was more sensitive to certain chemotherapy regimens and immunotherapy, providing informational clues for application of circadian gene signatures in clinical settings.

Coincidentally, circadian oscillation plays an important role in tumor-immune interaction, including antigen presentation, immunogenicity enhancement or loss and TME biology, during which these pathways might experience upregulation or downregulation depending on tumor type as well. When it comes to melanoma, higher CTL infiltration along with higher PD-1/L1 expression was found to be correlated with higher BMAL1 during anti-PD-1 immunotherapy clinical settings [45]. While in KIRC and breast cancer, upregulation of CLOCK, ARNTL and PER3 promotes TME inflammation via modulating macrophages and neutrophils infiltration, which leads to worse prognosis [46, 47]. However, the specific mechanisms under this myeloid-induced inflammation have not been demonstrated yet. In our research, high-risk patients’ TME is characterized as an immune-suppressed TME with more infiltration of macrophages, neutrophils, activated mast cells and activated DCs, while TME in low-risk groups has more CD8 T cells, activated CD4 Tm and rested masted cells, indicating a myeloid-dominated signature in high-risk group with worse prognosis as well. To be noticed, reasons behind this might be attributed to enhanced expression of ICs. For example, RORγ agonists, which can activate BMAL1 transcription [48], attenuate the expression of PD-1 receptors, and LYC-55716, a RORy inhibitor, is currently under a Phase 1 trial used in combination with pembrolizumab for NSCLC (NCT03396497). Nevertheless, direct and indirect circadian control over specific immune cells subtypes should be investigated thoroughly for their potential translational clinical applications.

Besides, PPI interaction built upon hub genes (CCL20, KRT16, GJB2) in our analysis pointed to interaction network among IL1A, CXCL1, CXCL8, S100A7 and S100A9, which are important mediators of myeloid immune cells function. The chemokine CCL20 is notably overexpressed by myeloid cells, as is its cognate CCR6 receptor on T cells. Disruption of the CCL20-CCR6 axis in mice restores CTL activity and significantly prolongs survival [32]. CXCL8 functions as a chemotactic factor by guiding the neutrophils to the site of infection [37]. IL1A is produced by monocytes and macrophages as a proprotein, which is proteolytically processed and released in response to cell injury, and thus induces apoptosis. Upregulated S100A7 could promote tumor proliferation through paracrine interaction with RAGE receptors [39]. To be noticed, through KEGG analysis we also uncovered RAGE receptor pathways enrichment in DEGs between high and low-risk groups. In addition, exocrine S100A7 could promote M2 macrophage infiltration in esophageal squamous carcinoma (ESCC) [36, 39]. SY Lim et al. demonstrated that monocytes/macrophages in the metastatic liver microenvironment induce S100A8 and S100A9 in cancer cells, and that these proteins are essential for tumor cell migration and invasion [36].

Another major cancer hallmark, metabolism disorder, is indispensable for cancer cells proliferation requirements under TME selection pressure as well. Constitutive activation of the PI3K/PDK1/AKT pathway and HIF1a pathway under low level of oxygen contribute to increased glycolysis in tumor [49]. Circadian clock has been proved to be essential regulators of glycolysis and oxidative phosphorylation through AKT and HIF1a pathways either [50]. Conversely, hypoxia and HIF1α affect circadian rhythms through regulation of the circadian clock genes CRYs, RORα, Per2, and Cry1 [51]. We also discovered enhanced expression pattern of fructose and mannose metabolism through KEGG analysis, as well as pentose phosphate and galactose metabolism pathways through GSEA analysis in high-risk groups of cervical cancer patients, further proving the potential link of glycolysis and circadian rhythm in cancer. Besides, PPAR signaling pathway and FA metabolism were also enriched in high-risk group, indicating regulation of FA metabolism by circadian oscillation as well. Recently, T Fedchenko et al. found that PPAR-γ agonist given to mice orally induced disruption of PER1/2 and BMAL1 expression in liver through regulation of NFKB and IL-6 pathways [52]. At the same time, PPAR signaling pathway is the center of de novo synthesis of fatty acids. Sai Ma et al. characterized a mutual activation loop between PPARγ and esophageal adenocarcinoma-specific master regulator transcription factors (MRTF) in upregulation of synthesis of phospholipids [53]. However, whether circadian clock signature could manipulate FA metabolism through PPAR pathways in cancer still lacks research till now.

Circadian rhythm interference has been attributed to uncontrolled proliferation and dampened DNA damage response, which are further linked to TMB and MSI. Emerging evidence have proved effects of circadian signature upon TMB and MSI, such as the positive regulation of growth promoter SERPINE1 via BMAL2, which promotes MSI [54]. Bioinformatics analysis of TCGA discovered an index of core circadian genes (PER1/2/3, CRY1/2, CLOCK and BMAL1) that is negatively related to MMR pathway [55]. However, we did not recognize significant TMB and MSI changes between high-risk and low-risk groups based upon circadian signature classification model in TCGA-CESC. Indeed, cancer heterogeneity and tissue origin specificity could make regulation of circadian clock even more complicated, indicating necessity for thorough investigation of circadian rhythm in different cancer type separately.

CCL20 was indicated as inducer of dampened anti-tumor ability of CTL when it was secreted by macrophages. We not only uncovered connection between expression of CCL20 and worse stage in clinical settings, but also uncovered disrupted CD8^+^ T cells function with exhaustion phenotype and M2 infiltration triggered by ccl20 overexpression in U14 cancer cell lines in immune-competent murine tumor model. Although Hirotaka et al. found that dietary consumption of Lactobacillus-derived exopolysaccharide induced CCR6^+^ CD8^+^ T cells by CCL20-secreting tumor cells [56], Wang et al. also uncovered FOXO1 promoted the migration of M2 macrophages via CCL20 secretion in esophageal squamous cell carcinoma [57]. Besides, resently Liu et al.revealed a specific M2-like macrophages subtype with high CCL20 expression, which is associated with worse prognosis by single-cell analysis in CESC [58]. Besides, they proposed that CCL20^+^ macrrophges also expressed high levels of CXCL8, which is in accordance with our PPI analysis in TCGA-CESC as well [59]. All in all, the regulation of CCL20 on the tumor immune microenvironment is currently controversial, our research might have shed light upon an indispensable role for M2 macrophages as well in cervical cancer.

Despite the critical role of CCL20 in our circadian-based risk model, KRT24, an OS-favorable protective marker in CESC has also been included into our model. KRT24 has also been proved to be a potential tumor suppressor. Désirée. et al. described suppression of viability and proliferation induced by KRT24 upon human HNSCC cell lines and mouse xenograft model [60]. However, the impact of KRT24 upon TME in CESC still lacks certain research. Based upon a significant up-regulated immune-score calculated by ssGSEA with more KRT24 expression (Fig. 11A), we proposed that in-depth profiling of impacts of KRT24 upon TME in CESC, as well as its correlation with CCL20 is in urgent need.

To our knowledge, this is the first comprehensive bioinformatics analysis of circadian rhythm signatures in cervical cancer. Through multi-omics analysis of TCGA-CESC dataset and using GSE44001 as verification, we successfully constructed a prognostic risk model based upon circadian rhythm signature and discovered three independent prognostic factors, GJB2, CCL20 and KRT24, with hints upon metabolism features and suppressive myeloid cells enriched TME as poor prognostic indicators. However, due to intrinsic nature of data-mining, the underlying mechanism of impacts of circadian clocks upon macrophages and neutrophils infiltration as well as PPAR signaling pathways and glycolysis-related pathways enrichment still need to be thoroughly verified by molecular and cell biology experiments further. Nevertheless, we believed that our research could facilitate understanding of clinical value of circadian rhythm in cervical cancer, and unleash probability of digging into the prognostic value and even targetable features of circadian clock gene sets in cancer biology.

## Conclusions

Circadian clock disruption has been proved to relate to cancer progression. However, its relevant significance in cervical cancer still lacks thorough research. Through multi-omics analysis of TCGA and GEO publicly available data, we built up a circadian-clock signature based prognostic model, with GJB2, CCL20 and KRT24 as independent-significant prognostic factor. Further through CIBERSORT analysis, FISH and IHC analysis of clinical specimens and flow cytometry analysis of subcutaneous mouse tumor model, CCL20 was identified as inducer of desert-like TME with more M2 macrophages infiltration and as indicator of worse clinical stages.

## Supplementary Information

** Additional file 1: Figure S1.** Differential gene analysis of circadian subgroups. (A) is the dotplot of expression of selected circadian genes between GEO-collected normal cervical tissues (101) and GTEx normal cervical tissues (13) (upper), and integration analysis of normal cervical tissues (114) compared with TCGA-CESC tumor tissues (304) (down); (B) is the heatmap of circadian candidate genes from a validation dataset GSE9750 (normal=23,tumor=33); (C). Differential gene heatmap between high and low-risk groups; (D). Differential gene volcano map between high and low-risk groups. **Figure S2.** Prognostic value of KRT24 in cases of OS or PFS. (A) is time-dependent AUC analysis of KRT24 in OS, with each broken line represents the change of the AUC value at the indicated time point; (B) is time-dependent AUC analysis of KRT24 in PFS; (C) is time-dependent ROC analysis of KRT24 for 1, 3 and 5 years’ PFS. **Figure S3.** (A) Circadian gene expression between high and low risk groups; (B) is the survival plot of patient separated by the risk model in TCGA-CESC; (C-D) are multivariate cox analysis of OS and disease free survival (DSS) of patients in TCGA-CESC by TNM stages, CCL20, KRT24 and GNB2. **Figure S4.** PPI network of core genes derived from transcriptome DEG analysis between high-risk and low-risk groups. **Figure S5.** Validation of CCL20-OE U14 cell lines.(A) Immunocytochemistry (ICC) of CCL20 expression for U14 cell lines of NC (NC-CCL20) (left) or overexpression (OE-CCL20) (right); (B) is western blot (WB) plot of NC (left) or OE (right), with HSP90 as the loading control; (C) is the flow cytometry plot of NC-CCL20 (left) or OE-CCL20 (right); (D) is the qRT-PCR results of CCL20 with blue indicating NC-CCL20 and red indicading OE-CCL20. **Table S1. **Baseline data of TCGA-CESC patients in TCGA database. **Table S2. **CNVs of 24 circadian gene signatures in TCGA-CESC. **Table S3. **Top20 significant GO analysis. **Table S4. **Top20 significant KEGG analysis. **Table S5.**GSEA analysis results.

## Data Availability

All data associated with this study are present in the paper or the Supplementary Materials. After publication, all reasonable requests for materials, data, and code will be fulfilled after completion of a material transfer agreement between the International Peace Maternity and Child Health Hospital, School of Medicine, Shanghai Jiao Tong University.

## Abbreviations

ZT: Zeitgeber time
PCA: Principal component analysis
GO: Gene ontology
BP: Biological process
MF: Molecular function
CC: Cellular component
KEGG: Kyoto encyclopedia of genes and genomes
SNP: Single nucleotide polymorphism
FISH: Fluorescence in situ hybridization
FFPE: Formalin-fixed, paraffin-embedded
IHC: Immunohistochemistry
HRP: Horseradish peroxidase
DAPI: 4′-6′-Diamidino-2-phenylindole
FBS: Fetal bovine serum
MOI: Multiplicity of infection
SPF: Specific-pathogen-free
ROC: Receiver operating characteristic
AUC: Area under the curve
CNV: Copy number variations
SNP: Single nucleotide polymorphisms
TLR: Toll-like receptors
