# Correction to: Construction of a CCL20-centered circadian-signature based prognostic model in cervical cancer

**DOI:** 10.1186/s12935-023-02968-w

**Published:** 2023-06-23

**Authors:** Yuchong Yu, Yao Liu, Yuhong Li, Xiaoming Yang, Mi Han, Qiong Fan

**Affiliations:** 1 Department of Gynecologic Oncology, The International Peace Maternity and Child Health Hospital, School of Medicine, Shanghai Jiao Tong University, Shanghai, China; 2 Shanghai Municipal Key Clinical Specialty of Gynecologic Oncology, Shanghai, China; 3 Shanghai Key Laboratory of Embryo Original Diseases Affiliated to Shanghai Jiao Tong University School of Medicine, Shanghai, China

**Correction to: Cancer Cell International (2023) 23:92**

10.1186/s12935-023-02926-6

In this article Yuchong Yu and Yao Liu should have been denoted as co-first authors and they contributed equally to this article.

