# Supplementary material for: Construction of a CCL20-centered circadian-signature based prognostic model in cervical cancer
Source: Cancer Cell Int. 2023 May 15;23:92. doi: 10.1186/s12935-023-02926-6 (PMC10184429; doi:10.1186/s12935-023-02926-6)
Supplement: Supplementary file 1 — Additional file 1: Figure S1. Differential gene analysis of circadian subgroups. (A) is the dotplot of expression of selected circadian genes between GEO-collected normal cervical tissues (101) and GTEx normal cervical tissues (13) (upper), and integration analysis of normal cervical tissues (114) compared with TCGA-CESC tumor tissues (304) (down); (B) is the heatmap of circadian candidate genes from a validation dataset GSE9750 (normal=23,tumor=33); (C). Differential gene heatmap between high and low-risk groups; (D). Differential gene volcano map between high and low-risk groups. Figure S2. Prognostic value of KRT24 in cases of OS or PFS. (A) is time-dependent AUC analysis of KRT24 in OS, with each broken line represents the change of the AUC value at the indicated time point; (B) is time-dependent AUC analysis of KRT24 in PFS; (C) is time-dependent ROC analysis of KRT24 for 1, 3 and 5 years’ PFS. Figure S3. (A) Circadian gene expression between high and low risk groups; (B) is the survival plot of patient separated by the risk model in TCGA-CESC; (C-D) are multivariate cox analysis of OS and disease free survival (DSS) of patients in TCGA-CESC by TNM stages, CCL20, KRT24 and GNB2. Figure S4. PPI network of core genes derived from transcriptome DEG analysis between high-risk and low-risk groups. Figure S5. Validation of CCL20-OE U14 cell lines.(A) Immunocytochemistry (ICC) of CCL20 expression for U14 cell lines of NC (NC-CCL20) (left) or overexpression (OE-CCL20) (right); (B) is western blot (WB) plot of NC (left) or OE (right), with HSP90 as the loading control; (C) is the flow cytometry plot of NC-CCL20 (left) or OE-CCL20 (right); (D) is the qRT-PCR results of CCL20 with blue indicating NC-CCL20 and red indicading OE-CCL20. Table S1. Baseline data of TCGA-CESC patients in TCGA database. Table S2. CNVs of 24 circadian gene signatures in TCGA-CESC. Table S3. Top20 significant GO analysis. Table S4. Top20 significant KEGG analysis. Table S5.GSEA analysis results. [file 12935_2023_2926_MOESM1_ESM.docx]

***Additional Material***

**Additional Figures and Tables**

**Additional Figures**

**
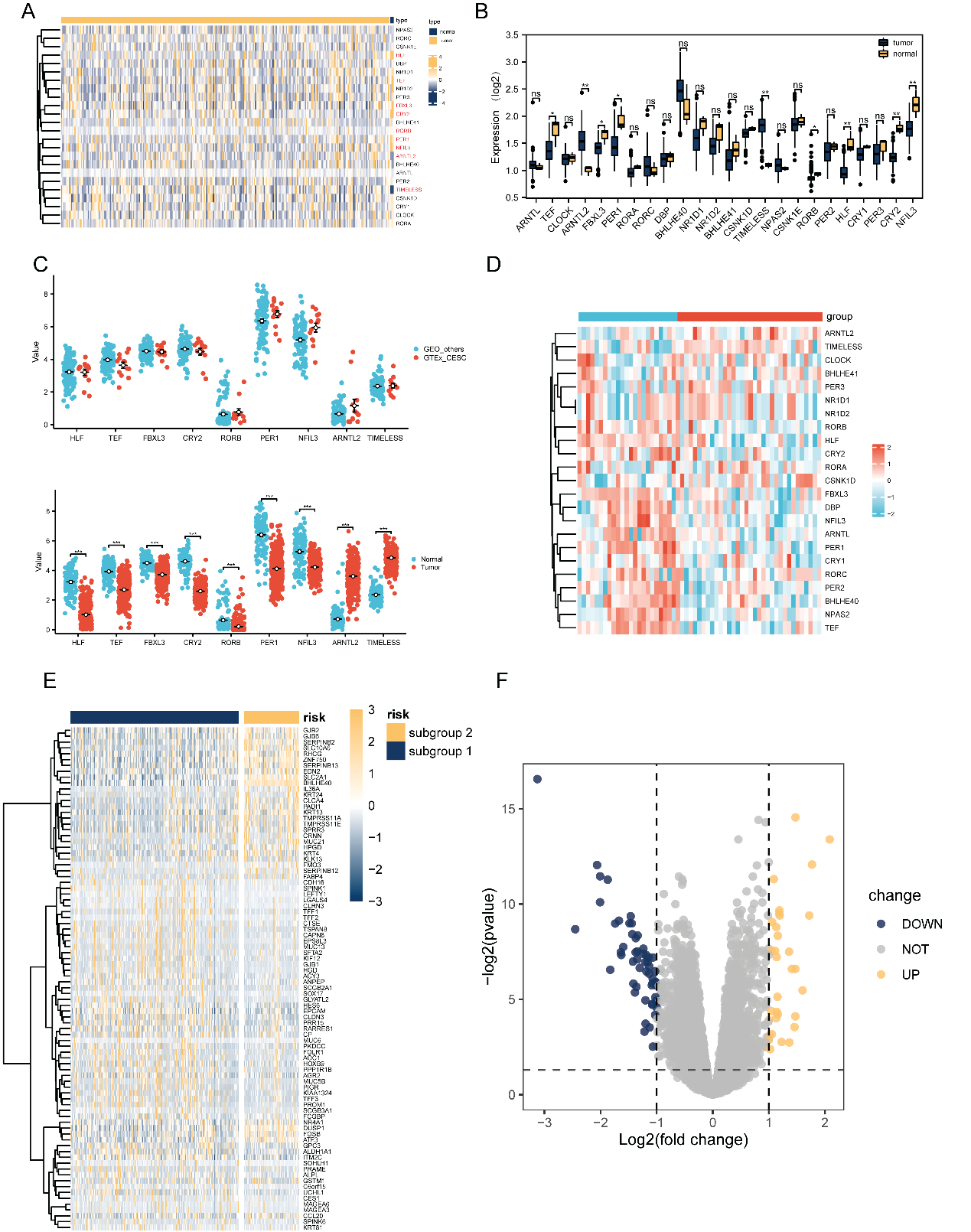
**

**Additional Figure 1.** Differential gene analysis of circadian subgroups. (A) heat map of differential expression of circadian rhythm genes in normal group (13) and tumor group (304) ; (B) box plot of differential expression of circadian rhythm genes in normal group (13) and tumor group (304); (C) is the dotplot of expression of selected circadian genes between GEO-collected normal cervical tissues (101) and GTEx normal cervical tissues (13) (upper), and integration analysis of normal cervical tissues (114) compared with TCGA-CESC tumor tissues (304) (down); (D) is the heatmap of circadian candidate genes from a validation dataset GSE9750 (normal=23,tumor=33); (E). Differential gene heatmap between high and low-risk groups; (F). Differential gene volcano map between high and low-risk groups.


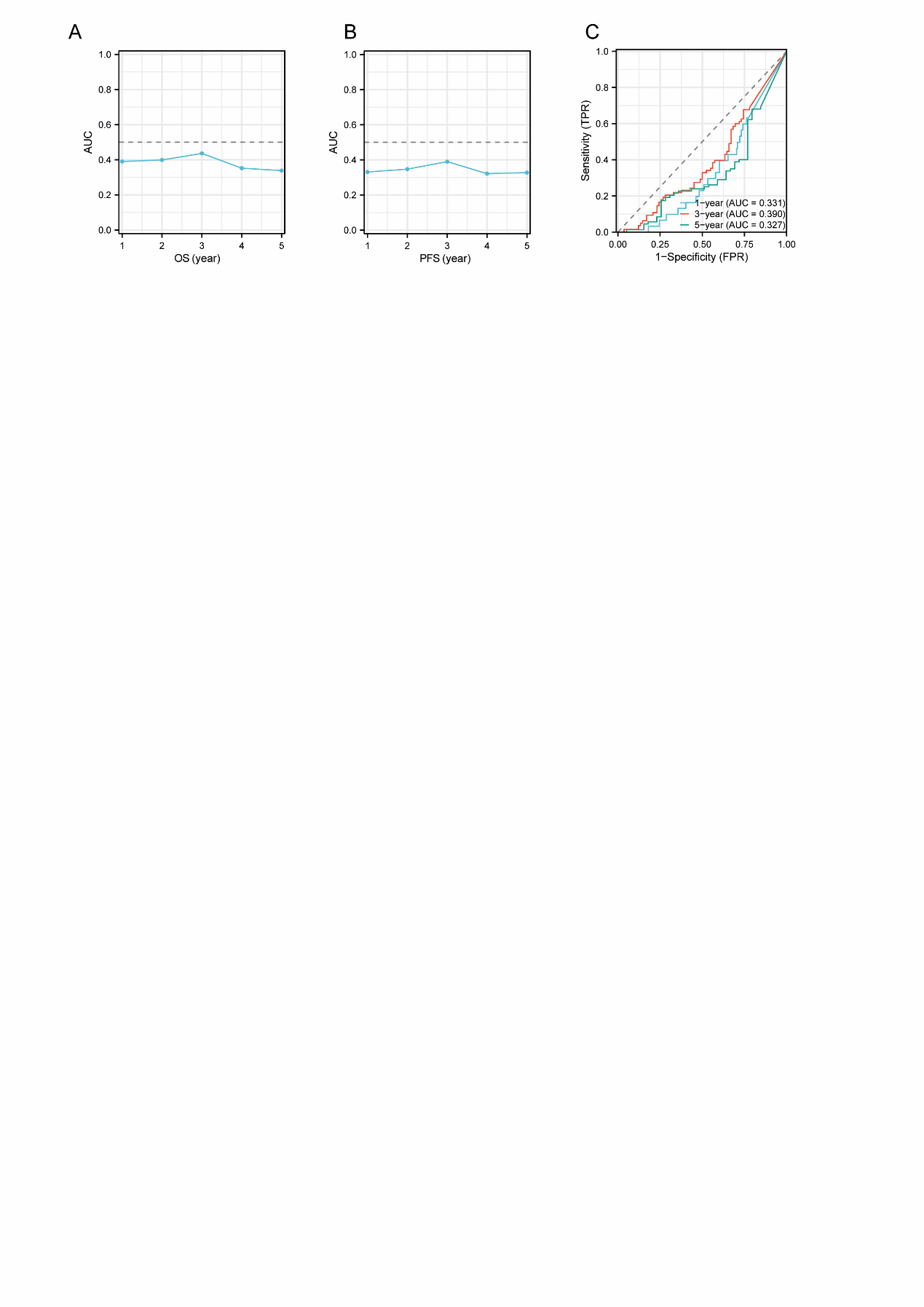


**Additional Figure 2.** Prognostic value of KRT24 in cases of OS or PFS. (A) is time-dependent AUC analysis of KRT24 in OS, with each broken line represents the change of the AUC value at the indicated time point; (B) is time-dependent AUC analysis of KRT24 in PFS; (C) is time-dependent ROC analysis of KRT24 for 1, 3 and 5 years’ PFS.


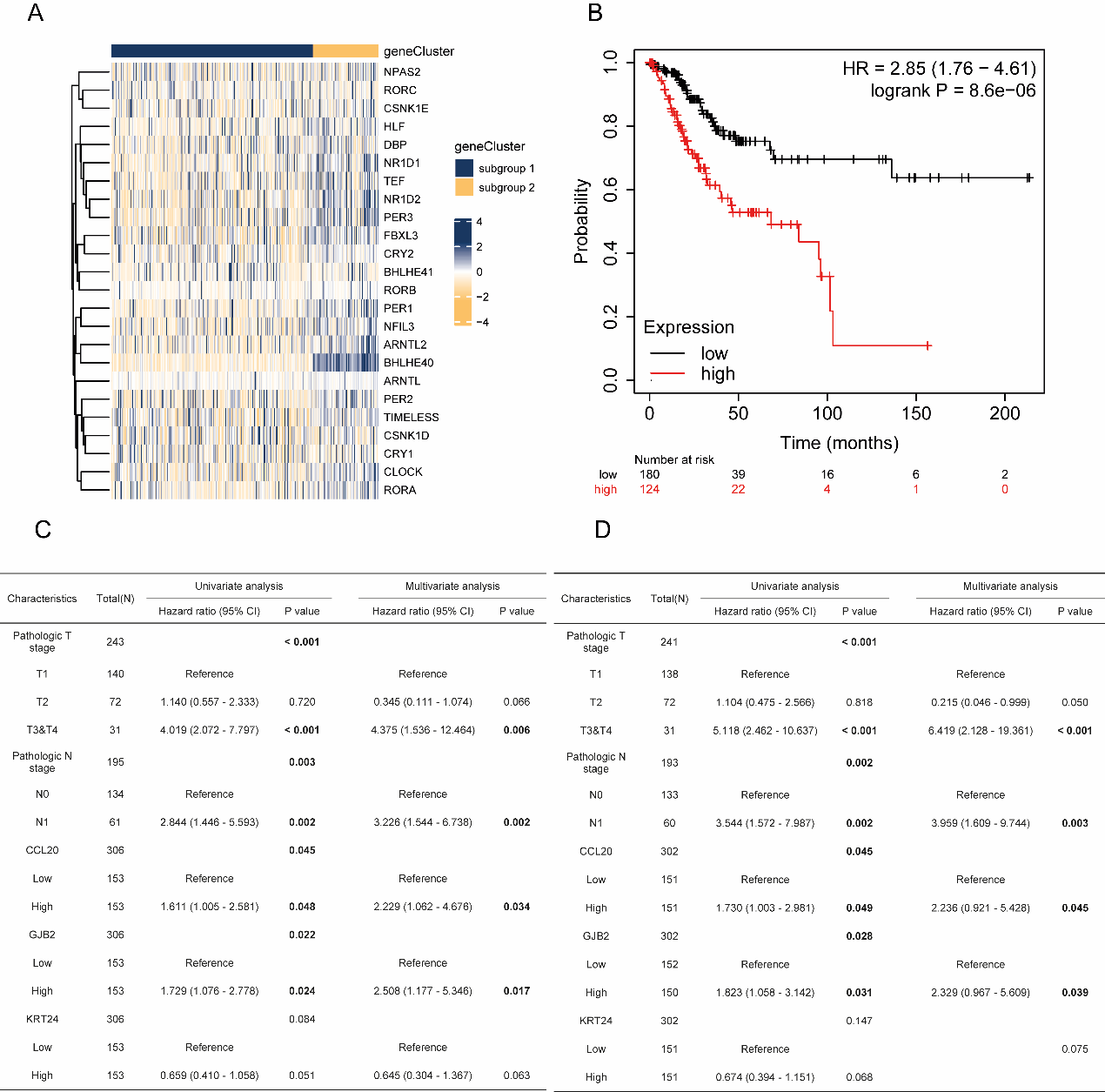


**Additional Figure 3.** (A) Circadian gene expression between high and low risk groups; (B) is the survival plot of patient separated by the risk model in TCGA-CESC; (C-D) are multivariate cox analysis of OS and DSS (disease free survival) of patients in TCGA-CESC by TNM stages, CCL20, KRT24 and GNB2.


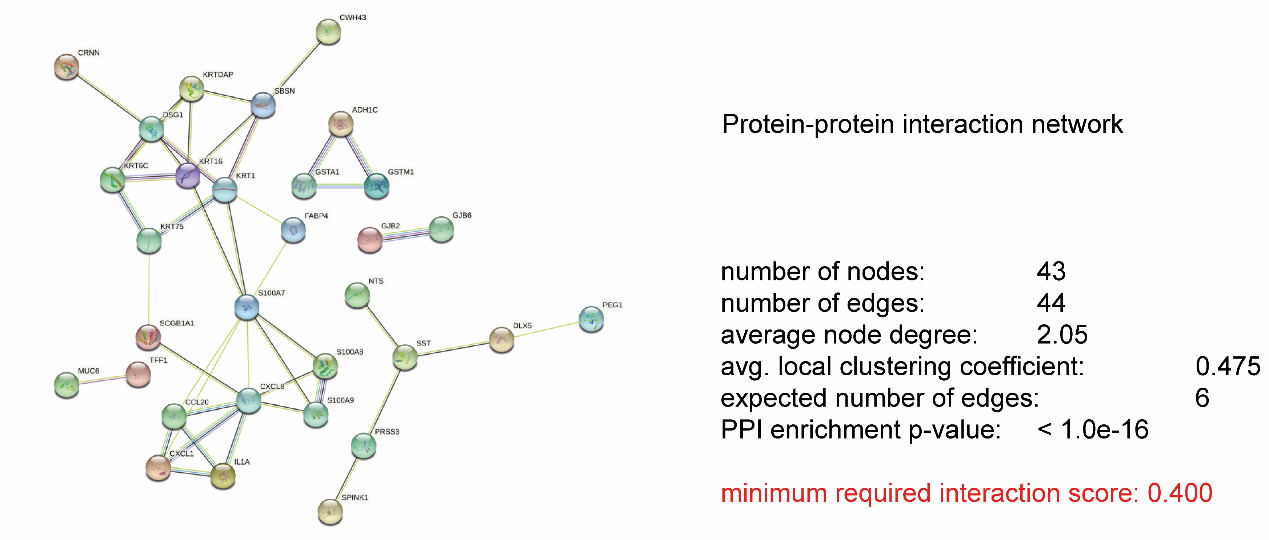


**Additional Figure 4.** PPI network of core genes derived from transcriptome DEG analysis between high-risk and low-risk groups.


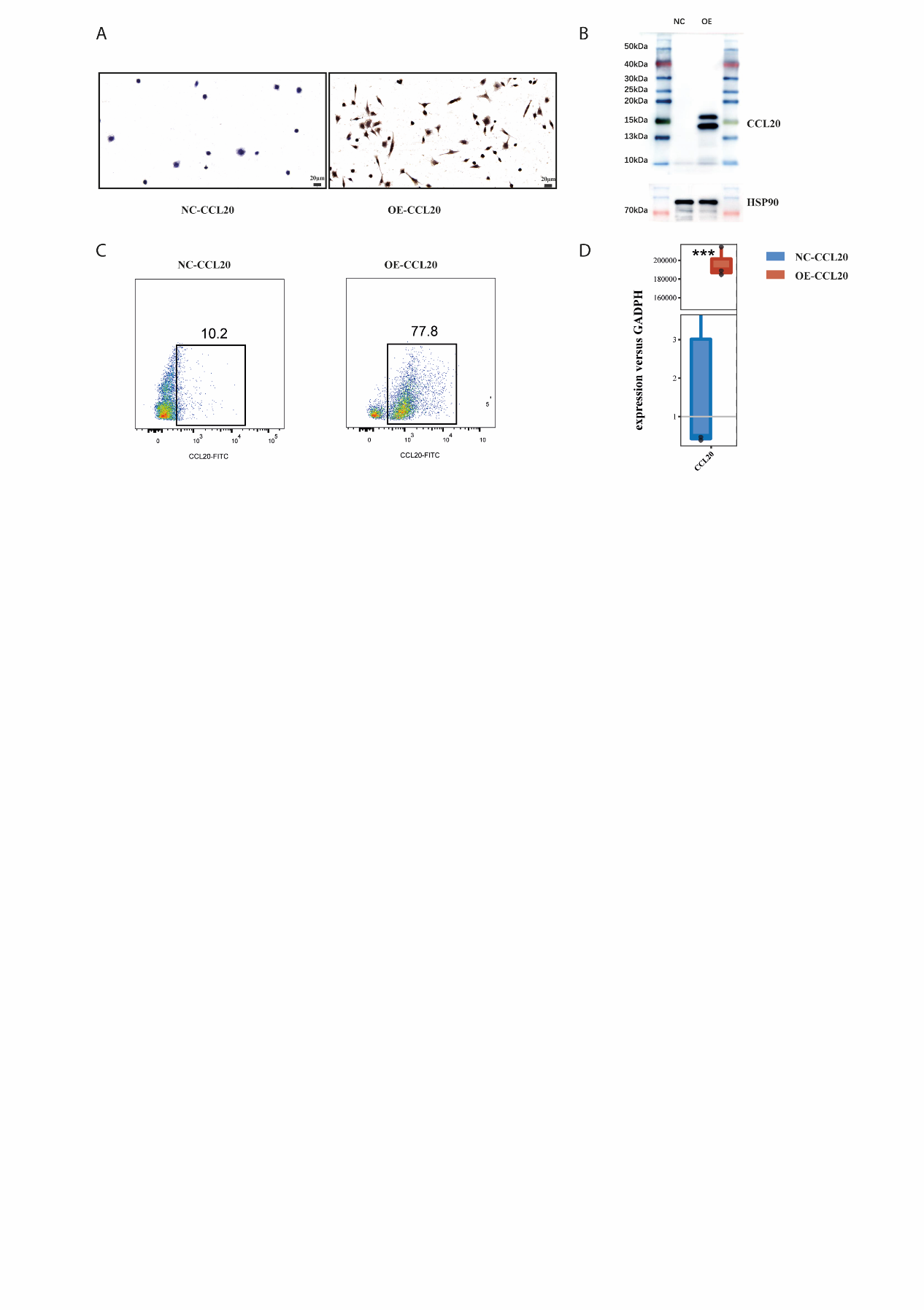


**Additional Figure 5.**Validation of CCL20-OE U14 cell lines.(A) Immunocytochemistry (ICC) of CCL20 expression for U14 cell lines of NC (NC-CCL20) (left) or overexpression (OE-CCL20) (right); (B) is western blot (WB) plot of NC (left) or OE (right), with HSP90 as the loading control; (C) is the flow cytometry plot of NC-CCL20 (left) or OE-CCL20 (right); (D) is the qRT-PCR results of CCL20 with blue indicating NC-CCL20 and red indicading OE-CCL20.

**Additional Tables**

| **Characteristic** | **levels** | **Overall** |
| --- | --- | --- |
| n |  | 279 |
| Age, n (%) | <60 | 221 (79.2%) |
|  | >=60 | 58 (20.8%) |
| pStage, n (%) | Miss | 6 (2.2%) |
|  | Stage I | 150 (53.8%) |
|  | Stage II | 63 (22.6%) |
|  | Stage III | 39 (14%) |
|  | Stage IV | 21 (7.5%) |
| event, n (%) | Alive | 216 (77.4%) |
|  | Death | 63 (22.6%) |
| survtime, n (%) | <5 year | 239 (85.7%) |
|  | >=5 year | 40 (14.3%) |

**Table S1** Baseline data of TCGA-CESC patients in TCGA database

| **symbol** | **a_total** | **d_total** | **a_hete** | **d_hete** | **a_homo** | **d_homo** | **entrez** |
| --- | --- | --- | --- | --- | --- | --- | --- |
| ARNTL | 4.40678 | 34.57627 | 4.40678 | 34.57627 | 0 | 0 | 406 |
| ARNTL2 | 18.64407 | 13.89831 | 17.9661 | 13.55932 | 0.677966 | 0.338983 | 56938 |
| BHLHE40 | 12.54237 | 36.27119 | 11.86441 | 35.25424 | 0.677966 | 1.016949 | 8553 |
| BHLHE41 | 18.64407 | 13.89831 | 17.9661 | 13.55932 | 0.677966 | 0.338983 | 79365 |
| CLOCK | 6.779661 | 27.45763 | 5.762712 | 27.45763 | 1.016949 | 0 | 9575 |
| CRY1 | 15.9322 | 7.457627 | 15.9322 | 7.457627 | 0 | 0 | 1407 |
| CRY2 | 6.779661 | 27.79661 | 6.101695 | 27.45763 | 0.677966 | 0.338983 | 1408 |
| CSNK1D | 21.35593 | 15.9322 | 21.01695 | 15.25424 | 0.338983 | 0.677966 | 1453 |
| CSNK1E | 11.86441 | 24.40678 | 11.52542 | 24.40678 | 0.338983 | 0 | 1454 |
| DBP | 28.47458 | 12.20339 | 27.45763 | 12.20339 | 1.016949 | 0 | 1628 |
| FBXL3 | 17.9661 | 25.42373 | 16.27119 | 25.08475 | 1.694915 | 0.338983 | 26224 |
| HLF | 20.67797 | 9.152542 | 20 | 9.152542 | 0.677966 | 0 | 3131 |
| NFIL3 | 17.62712 | 18.64407 | 17.28814 | 18.30508 | 0.338983 | 0.338983 | 4783 |
| NPAS2 | 12.88136 | 10.84746 | 12.20339 | 10.84746 | 0.677966 | 0 | 4862 |
| NR1D1 | 15.59322 | 11.18644 | 14.91525 | 11.18644 | 0.677966 | 0 | 9572 |
| NR1D2 | 11.18644 | 37.9661 | 10.84746 | 37.28814 | 0.338983 | 0.677966 | 9975 |
| PER1 | 4.745763 | 36.61017 | 4.745763 | 36.61017 | 0 | 0 | 5187 |
| PER2 | 4.067797 | 41.69492 | 3.728814 | 37.62712 | 0.338983 | 4.067797 | 8864 |
| PER3 | 27.79661 | 13.22034 | 27.79661 | 12.20339 | 0 | 1.016949 | 8863 |
| RORA | 18.64407 | 18.64407 | 18.64407 | 18.30508 | 0 | 0.338983 | 6095 |
| RORB | 16.94915 | 18.64407 | 16.94915 | 17.9661 | 0 | 0.677966 | 6096 |
| RORC | 53.22034 | 2.372881 | 50.16949 | 2.372881 | 3.050848 | 0 | 6097 |
| TEF | 10.84746 | 27.11864 | 10.16949 | 26.44068 | 0.677966 | 0.677966 | 7008 |
| TIMELESS | 17.28814 | 5.423729 | 16.27119 | 5.423729 | 1.016949 | 0 | 8914 |

**Table S2.** CNVs of 24 circadian gene signatures in TCGA-CESC

| **class** | **ID** | **Descrption** | **Pvalue** | **Qvalue** |
| --- | --- | --- | --- | --- |
| Cellular Component | GO:0005576 | extracellular region | 1.54E-14 | 2.52E-12 |
| Cellular Component | GO:0044421 | extracellular region part | 7.56E-13 | 6.20E-11 |
| Cellular Component | GO:0005615 | extracellular space | 1.21E-12 | 6.64E-11 |
| Cellular Component | GO:0031982 | vesicle | 1.34E-07 | 5.48E-06 |
| Cellular Component | GO:0070062 | extracellular exosome | 3.43E-07 | 9.47E-06 |
| Cellular Component | GO:1903561 | extracellular vesicle | 3.99E-07 | 9.47E-06 |
| Cellular Component | GO:0043230 | extracellular organelle | 4.04E-07 | 9.47E-06 |
| Biological Process | GO:0032496 | response to lipopolysaccharide | 3.02E-08 | 3.34E-05 |
| Biological Process | GO:0002237 | response to molecule of bacterial origin | 4.77E-08 | 3.34E-05 |
| Biological Process | GO:0070268 | cornification | 1.52E-07 | 7.11E-05 |
| Biological Process | GO:0009617 | response to bacterium | 2.15E-07 | 7.54E-05 |
| Biological Process | GO:0071621 | granulocyte chemotaxis | 2.79E-07 | 7.81E-05 |
| Biological Process | GO:0030855 | epithelial cell differentiation | 6.54E-07 | 0.0001348 |
| Biological Process | GO:0019730 | antimicrobial humoral response | 6.74E-07 | 0.0001348 |
| Biological Process | GO:0097530 | granulocyte migration | 7.91E-07 | 0.0001385 |
| Molecular Function | GO:0050786 | RAGE receptor binding | 1.22E-06 | 0.0001561 |
| Molecular Function | GO:0005504 | fatty acid binding | 1.83E-06 | 0.0001561 |
| Biological Process | GO:0042119 | neutrophil activation | 1.04E-06 | 0.000162 |
| Biological Process | GO:0036230 | granulocyte activation | 1.17E-06 | 0.0001636 |
| Biological Process | GO:0006959 | humoral immune response | 1.47E-06 | 0.0001695 |

**Table S3** Top20 significant GO analysis

| **Pathway ID** | **KEGG_class** | **Pathway** | **Pvalue** |
| --- | --- | --- | --- |
| ko04657 | Immune system | IL-17 signaling pathway | 1.86E-07 |
| ko00982 | Xenobiotics biodegradation and metabolism | Drug metabolism - cytochrome P450 | 0.0008978 |
| ko05323 | Immune diseases | Rheumatoid arthritis | 0.0010327 |
| ko00980 | Xenobiotics biodegradation and metabolism | Metabolism of xenobiotics by cytochrome P450 | 0.0010976 |
| ko05204 | Cancers | Chemical carcinogenesis | 0.0016308 |
| ko04061 | Signaling molecules and interaction | Viral protein interaction with cytokine and cytokine receptor | 0.0025847 |
| ko04915 | Endocrine system | Estrogen signaling pathway | 0.0065999 |
| ko05418 | Cardiovascular diseases | Fluid shear stress and atherosclerosis | 0.0067302 |
| ko04060 | Signaling molecules and interaction | Cytokine-cytokine receptor interaction | 0.0087577 |
| ko05134 | Infectious diseases | Legionellosis | 0.0110204 |
| ko05150 | Infectious diseases | Staphylococcus aureus infection | 0.0115547 |
| ko00480 | Metabolism of other amino acids | Glutathione metabolism | 0.0121393 |
| ko05164 | Infectious diseases | Influenza A | 0.0126692 |
| ko05120 | Infectious diseases | Epithelial cell signaling in Helicobacter pylori infection | 0.0157805 |
| ko04062 | Immune system | Chemokine signaling pathway | 0.0163765 |
| ko01524 | Drug resistance | Platinum drug resistance | 0.017982 |
| ko04971 | Digestive system | Gastric acid secretion | 0.0188972 |
| ko05133 | Infectious diseases | Pertussis | 0.0193621 |
| ko00983 | Xenobiotics biodegradation and metabolism | Drug metabolism - other enzymes | 0.0203045 |
| ko04933 | Endocrine and metabolic diseases | AGE-RAGE signaling pathway in diabetic complications | 0.0324866 |

**Table S4** Top20 significant KEGG analysis

| **Description** | **setSize** | **NES** | **pvalue** | **FDR** |
| --- | --- | --- | --- | --- |
| KEGG_NOD_LIKE_RECEPTOR_SIGNALING_PATHWAY | 61 | 2.06 | 2.97E-03 | 6.72E-02 |
| KEGG_FRUCTOSE_AND_MANNOSE_METABOLISM | 33 | 1.74 | 1.16E-02 | 1.16E-01 |
| KEGG_DRUG_METABOLISM_OTHER_ENZYMES | 51 | 1.71 | 9.06E-03 | 1.00E-01 |
| KEGG_PPAR_SIGNALING_PATHWAY | 69 | 1.69 | 6.17E-03 | 8.63E-02 |
| KEGG_OLFACTORY_TRANSDUCTION | 373 | 1.66 | 5.88E-03 | 8.63E-02 |
| KEGG_PENTOSE_PHOSPHATE_PATHWAY | 27 | 1.56 | 3.12E-02 | 1.99E-01 |
| KEGG_GALACTOSE_METABOLISM | 26 | 1.55 | 3.06E-02 | 1.98E-01 |
| KEGG_RIG_I_LIKE_RECEPTOR_SIGNALING_PATHWAY | 69 | 1.53 | 1.85E-02 | 1.51E-01 |
| KEGG_HISTIDINE_METABOLISM | 28 | 1.49 | 5.10E-02 | 2.58E-01 |
| KEGG_EPITHELIAL_CELL_SIGNALING_IN_HELICOBACTER_PYLORI_INFECTION | 68 | 1.47 | 2.80E-02 | 1.89E-01 |
| KEGG_COMPLEMENT_AND_COAGULATION_CASCADES | 69 | 1.46 | 3.09E-02 | 1.98E-01 |
| KEGG_CYTOKINE_CYTOKINE_RECEPTOR_INTERACTION | 261 | 1.45 | 1.84E-02 | 1.51E-01 |
| KEGG_STARCH_AND_SUCROSE_METABOLISM | 51 | 1.43 | 3.93E-02 | 2.25E-01 |
| KEGG_TOLL_LIKE_RECEPTOR_SIGNALING_PATHWAY | 102 | 1.42 | 2.11E-02 | 1.62E-01 |
| KEGG_APOPTOSIS | 87 | 1.34 | 4.51E-02 | 2.40E-01 |
| KEGG_JAK_STAT_SIGNALING_PATHWAY | 155 | 1.27 | 3.42E-02 | 2.11E-01 |
| KEGG_TGF_BETA_SIGNALING_PATHWAY | 85 | -1.39 | 3.92E-02 | 2.25E-01 |
| KEGG_PROTEASOME | 43 | -1.39 | 5.40E-02 | 2.61E-01 |
| KEGG_T_CELL_RECEPTOR_SIGNALING_PATHWAY | 107 | -1.42 | 2.81E-02 | 1.89E-01 |
| KEGG_INTESTINAL_IMMUNE_NETWORK_FOR_IGA_PRODUCTION | 46 | -1.42 | 4.27E-02 | 2.32E-01 |

**Table S5** GSEA analysis results
